# Metastable π‐Lithium Supermolecule Entities Govern Voltage in Electron‐Rich Electrolytes: Orbital Energetics as Predictive Voltage Descriptors

**DOI:** 10.1002/advs.202517037

**Published:** 2025-11-23

**Authors:** Yiwei Feng, Hui Dong, Xinyu Song, Yuxiang Bu

**Affiliations:** ^1^ School of Chemistry and Chemical Engineering Shandong University Jinan 250100 P. R. China

**Keywords:** ab initio molecular dynamics, Ï€‐lithium supermolecule entity, lithium solvated electron solution, open‐circuit voltage, pull‐push hyper‐covalent interactions

## Abstract

Lithium‐solvated‐electron solutions (Li‐SESs) hold immense promise as transformative, high energy‐storage media for applications such as liquid batteries and anode prelithiation. However, their fundamental architectures and intrinsic voltage regulation mechanisms remain incompletely understood. Through ab initio molecular dynamics simulations, we find a novel class of electroactive Lithium‐p supermolecule entities (SMEs), which serve as key voltage‐modulating components. These SMEs form through synergistic aggregation of polycyclic aromatic hydrocarbons (PAHs), Li^+^, solvated‐electrons, and tetrahydrofuran where PAHs function as π‐scaffolds through hyperconjugation‐driven push‐pull interactions. We establish a robust open‐circuit voltage (OCV) computation model demonstrating strong linear correlations across 8 PAH‐Li‐SESs (R^2^〉0.96). Crucially, OCV correlates with the highest‐occupied‐molecular‐orbital energy of SME, intrinsically linked to the lowest‐unoccupied‐molecular‐orbital (LUMO) energy of PAH molecule, enabling a predictive PAH‐LUMO‐to‐OCV relationship. This dual‐descriptor framework achieves quantitative OCV predictions for 23 PAHs (including 15 previously unexplored systems), validated against experimental data. PAHs with LUMO‐energy〈 ‐2.0 eV consistently yield OCV 〉 900 mV, enabling rational design of high‐voltage anolytes. Consequently, we develop a high‐through put screening strategy using PAH LUMO‐energy to rapidly identify additives for high‐voltage Li‐SESs and electron‐rich electrolytes. Overall, this SME‐framework facilitates rational additive engineering for high‐voltage alkali‐metal SES anolytes and optimized prelithiation reagents, significantly accelerating advanced electrolyte development.

## Graphical abstract

Solvated electron supermolecules (SMEs) formed by polycyclic aromatic hydrocarbon (PAH), Li⁺, and tetrahydrofuranmodulate voltage in lithium solutions. The open‐circuit‐voltage (OCV) of such solutions correlates with the lowest unoccupied molecular orbital energy of PAH, enabling high‐throughput screening for high‐voltage electrolytes. This framework supports rational design of high‐performance anolytes and prelithiation reagents.

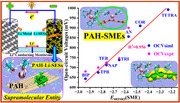

## 1 Introduction

Lithium‐ion batteries (LIBs), recognized as vital energy storage systems and honored with the 2019 Nobel Prize in Chemistry,^[^ ^1^, ^2^ ^]^ have been the focus of extensive research for decades.^[^ ^3^, ^4^, ^5^, ^6^, ^7^, ^8^, ^9^ ^]^ Although significant efforts are directed toward developing solid‐state LIBs with graphite anodes,^[^ ^10^, ^11^, ^12^ ^]^ broader adoption necessitates overcoming critical challenges, including high costs, slow charge/discharge rates, and safety concerns.^[^ ^8^, ^9^, ^13^ ^]^ A promising strategy to address these limitations involves employing liquid‐based metal electrodes, which offer high solubility for active species, facilitate rapid lithium cation (Li^+^) transport, eliminate phase deformation and grain size fluctuations, and prevent dendrite formation.^[^ ^14^ ^]^ However, challenges persist, such as susceptibility to corrosion, elevated operating temperatures, and high vapor pressure.^[^ ^14^ ^]^ Alternative approaches explore semi‐solid‐state electrode materials, encompassing semi‐solid flow cells^[^ ^15^, ^16^ ^]^ and liquid redox flow batteries.^[^ ^17^, ^18^, ^19^, ^20^ ^]^ Yet, redox flow batteries suffer from low specific energy density, while semi‐solid lithium flow cells exhibit poor conductivity, requiring substantial conductive additives.^[^ ^21^ ^]^ Consequently, highly concentrated liquid‐based electrode materials, separated by a Li^+^‐conducting membrane, represent a potential solution, aiming to merge the benefits of redox flow batteries and semi‐solid lithium flow cells.^[^ ^22^, ^23^ ^]^

Solvated electron solutions (SESs), particularly in anhydrous liquid tetrahydrofuran (THF), are highly promising as liquid‐state anode materials due to their exceptional electrochemical properties, including high open‐circuit voltages (OCVs) in fully liquid‐state cells.^[^ ^23^, ^33^ ^]^ From the perspective of practical rechargeable batteries, SESs are particularly significant because they enable stabilization and controllable distribution of solvated electrons in solution, which directly influences OCVs. Such unique features distinguish SESs from conventional electrolyte systems and highlight their potential for advancing next‐generation high‐performance rechargeable batteries. Lithium metal forms SESs (Li‐SESs) with various species, such as organic radicals, liquid ammonia, and polycyclic aromatic hydrocarbons (PAHs).^[^ ^31^, ^32^, ^33^ ^]^ PAHs, defined as hydrogen‐terminated subunits of 2D graphene sheets,^[^ ^34^ ^]^ dissolve in THF along with lithium metal to form PAH‐incorporated Li‐SESs (PAH‐Li‐SESs).^[^ ^25^, ^26^, ^27^, ^28^, ^29^ ^]^ These PAH‐Li‐SESs are exceptionally attractive: they stabilize solvated electrons (e^−^_sol_) and Li^+^ from dissolved lithium for pre‐lithiation;^[^ ^24^, ^33^ ^]^ they circumvent safety hazards associated with ammonia‐based systems;^[^ ^23^, ^24^ ^]^ they serve as excellent pre‐lithiation reagents for solid anodes;^[^ ^35^, ^36^ ^]^ and they exhibit excellent metallic‐like conductivity and electrochemical stability.^[^ ^24^, ^25^, ^26^, ^27^, ^28^, ^29^, ^30^, ^31^, ^32^, ^33^ ^]^ Furthermore, the structural resemblance of PAH additives to graphene subunits provides excellent electrochemical stability and tunability.

The performance of LIBs incorporating PAH‐Li‐SESs hinges critically on their structural compositions, binding characteristics, and electron distributions. Recent experimental evidence indicates that Li‐SESs utilizing naphthalene or biphenyl as additives exhibit the highest electrical conductivity, reaching ≈11 mS cm^−1^.^[^ ^25^, ^26^ ^]^ Concurrent efforts aim to enhance OCVs in Li‐SESs by modulating PAH size or incorporating heteroatoms.^[^ ^27^, ^28^, ^29^ ^]^ Selection of PAH additives typically considers structural parameters such as π‐conjugation length, molecular size, and electron affinity to balance solubility and application suitability (e.g., liquid anodes or pre‐lithiation reagents). Current screening strategies primarily rely on quantum chemistry calculations to evaluate binding energies and electronic properties of PAH‐Li complexes in solvents. While this work has advanced the understanding of OCVs in PAH‐Li‐SESs,^[^ ^25^, ^26^, ^27^, ^28^, ^29^, ^30^ ^]^ key mechanisms remain elusive. Specifically, the precise structures of the solvated PAH‐(Li^+^/e^−^)_n_ entities contributing to high OCVs, and the mechanistic role PAH additives play in governing OCVs and associated processes, require clarification. The performance of PAH‐Li‐SESs is fundamentally governed by solvated configurations dictated by solute‐solvent (PAH, Li) and solvent‐solvent interactions. Therefore, identifying optimal PAH additives and elucidating the interactions among Li^+^, e^−^_sol_, PAHs, and solvent molecules are paramount for achieving high OCVs in PAH‐Li‐SESs.

**Figure 1:**
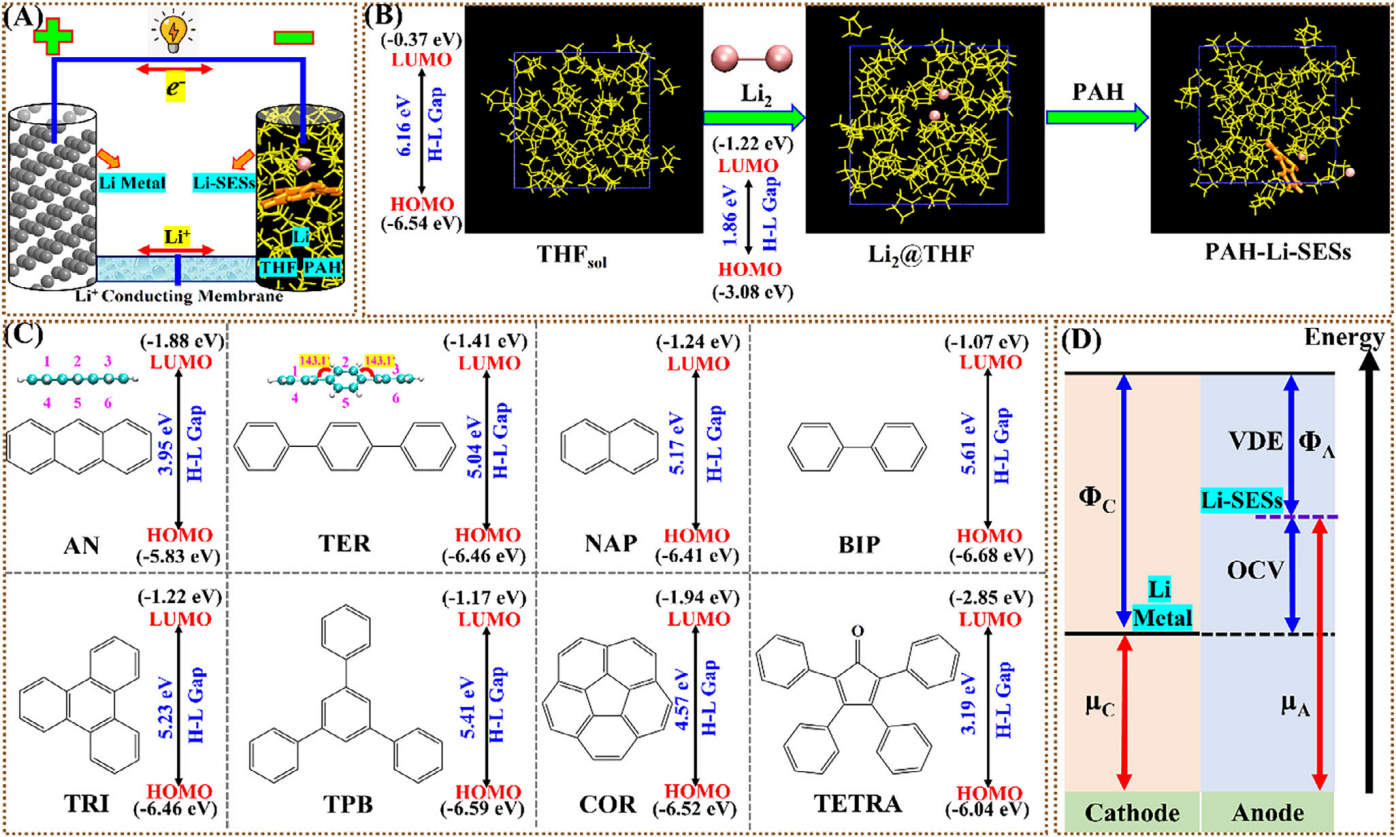
Cell structure and OCV model. A) Schematic diagram of the half‐cell working principle. B) Solvation model for AIMD simulations and transient snapshots of two solution systems (Li_2_@THF, PAH‐Li‐SESs). C) Molecular structures of PAH additives with their HOMO/LUMO energies and gaps (H‐L Gap). D) Proposed model to calculate OCV where µA and µ_C_ are the electronic chemical potentials of anode and reference cathode (here Li metal), and Φ_A_ and Φ_C_ are the work functions of two electrodes, respectively; Φ_A_ ≈ VDE (vertical detachment energy).

Anthracene (AN) and *p*‐terphenyl (TER) are representative PAH additives investigated previously, distinguished by their differing π‐conjugation structures (**Figure** **1**).^[^ ^37^, ^38^ ^]^ Recent studies on the electrochemical properties of Li‐SESs containing AN and TER in liquid THF confirm their role in stabilizing e^−^_sol_, and to a lesser extent, Li^+^ within Li‐SESs.^[^ ^27^, ^28^ ^]^ However, critical structural information for AN/TER‐Li‐SESs remains undiscovered, notably the binding modes between Li^+^, e^−^_sol_, and AN/TER, and the potential complex structures existing in THF solvation environments—factors likely crucial for achieving high OCVs. The solution structure is dynamic; putative complexes or chemical entities may exhibit structural variability, potentially transient or loosely bound, all influencing battery performance. Currently, there is scant understanding of the solvated structures of e^−^_sol_ and Li^+^ and their dynamics behavior in the presence of PAHs, or their specific impact on OCVs in liquid‐state LIBs using PAH‐Li‐SESs anodes. Deciphering these binding modes and interactions is essential for developing high‐performance liquid‐state anode materials. Thus, precisely characterizing solution structures and their dynamics, and uncovering their intrinsic influence on OCVs, is vital for optimizing battery performance and screening ideal additives.

Although experimental OCV determination typically employs half‐cell configurations, computational prediction presents significant theoretical challenges. Conventional models approximate OCVs via work function differences between electrodes. However, first‐principles calculations face inherent limitations in solid‐state systems: 3D periodic boundary conditions lack explicit vacuum regions, preventing accurate vacuum‐level determination—a prerequisite for work function derivation.^[^ ^39^, ^40^, ^41^ ^]^ This gap is particularly acute for Li‐SESs, where dynamic electroactive species invalidate static electrode assumptions. Existing frameworks are inadequate as they neglect solvent‐mediated quantum interactions and time‐dependent charge redistribution. Addressing this requires establishing a solution‐phase descriptor framework correlating OCVs with intrinsic electronic properties of solvated species, avoiding vacuum‐dependent approximations. Conversely, ab initio molecular dynamics (AIMD) simulations combined with density functional theory (DFT) calculations offer a powerful approach to investigating these complex solution dynamics. This methodology provides molecular‐ and electronic‐level insights into dynamic binding modes and their functional impacts, aligning well with experiment,^[^ ^42^, ^43^, ^44^ ^]^ and has been successfully applied to diverse systems.^[^ ^45^, ^46^, ^47^, ^48^, ^49^, ^50^ ^]^

Building upon this foundation, we construct a model Li‐SES anolyte containing formal lithium (supplying Li^+^ and e^−^) and PAHs (AN, TER, etc.) as co‐solvents in liquid THF, informed by experimental data.^[^ ^25^, ^26^, ^27^, ^28^, ^29^ ^]^ Specifically, AN, TER, naphthalene (NAP), biphenyl (BIP), triphenylene (TRI), and 1, 3, 5‐triphenylbenzenes (TPB) have demonstrated experimentally measured OCVs values when employed as liquid anode materials in Li‐SESs. In contrast, although corannulene (COR) and tetraphenylcyclopentadienones (TETRA) lack reported OCV measurements, their functionality as liquid anodes in Li‐SESs have been experimentally validated. Detailed experimental OCV data are presented in **Table** **1** Using integrated AIMD simulations and DFT calculations, we explore the structures within these Li‐SESs, focusing on the binding modes among Li^+^, e^−^_sol_, and PAHs in THF and predict their OCVs and other electronic properties (Figure 1). We identify a novel type of supermolecular structure entity (SME), [PAH‐Li]^−^(THF)_n_ (*n* = 1–2, PAH‐SME), which intrinsically governs the OCVs of Li‐SESs. Within these SMEs, the PAH acts as a shallow trap, holding 2 e^−^_sol_ and a Li^+^ through the incorporation of 1–2 THF molecules, mediated by dynamic pull‐push hyper‐covalent interactions. We introduce a novel model for computing PAH‐Li‐SES OCVs based on the vertical detachment energies (VDEs) of their constituent SMEs; predicted OCVs align closely with experimental values across six PAH‐Li‐SESs. Crucially, we establish a strong linear correlation between the predicted OCVs for all eight PAH‐Li‐SESs studied and either the highest occupied molecular orbital (HOMO) energy of their SMEs or the lowest unoccupied molecular orbital (LUMO) energy of the additive PAH molecule. This robust correlation underpins a new strategy for predicting LIB OCVs directly from the frontier orbital energies of SMEs or PAHs, facilitating efficient screening of high‐performance PAH additives, and demonstrating that both E_HOMO_(SME) and E_LUMO_(PAH) serve as reliable energetic descriptors. Beyond these experimentally benchmarked systems, an additional set of 15 PAHs without reported OCV values were further investigated, enabling a broader structural screening and predictive evaluation of Li‐SESs anode candidates. Employing this protocol, we predict the OCVs for 23 PAH additives; results exhibit strong agreement with experimental and AIMD‐simulated OCVs for the eight previously studied systems. Furthermore, we provide evaluative predictions for 15 additional PAH additives, identifying benzoquinone derivatives as yielding the highest OCVs (> 1100 mV). This work offers crucial dynamic insights into Li‐SESs and provides a new perspective for optimizing the binding patterns of formal lithium species (Li^+^, e^−^) in liquid‐state LIB anode materials. The SME concept proposed here is potentially generalizable to other liquid anodes or anolytes (e.g., sodium/potassium analogs), offering a novel paradigm for the exploration of next‐generation liquid batteries.

**Table 1:** *E*_HOMO_ of Supermolecule Entities (SMEs, E_HOMO_(SME), eV) and Simulated (OCV_siml_, mV) and Experimental (OCV_expt_, mV) OCVs for Li_2_@THF Solution and PAH‐Li‐SESs and LUMO Energies of PAH Molecules (*E*_LUMO_(PAH), eV). All OCVs referenced to metallic Li.

Symbols	SMEs	*E*_HOMO_[SME]	*E*_LUMO_[PAH]	OCV_siml_	OCV_expt_
Li_2_‐SME	(THF)_2.4_Li‐Li(THF)_0.7_	−2.14	–	632.1	–
TETRA‐SME	[TETRA‐Li]^−^(THF)_1_	−2.23	−2.85	992.3	–
COR‐SME	[COR‐Li]^−^(THF)_2_	−2.47	−1.94	910.5	–
AN‐SME	[AN‐Li]^−^(THF)_1_	−2.57	−1.88	846.4	900.0^[^ ^27^ ^]^
TRI‐SME	[TRI‐Li]^−^(THF)_1_	−2.65	−1.22	738.6	747.2^[^ ^56^ ^]^
TER‐SME	[TER‐Li]^−^(THF)_2_	−2.77	−1.41	718.4	725.0^[^ ^28^ ^]^
NAP‐SME	[NAP‐Li]^−^(THF)_2_	−2.76	−1.24	712.4	720.0^[^ ^26^ ^]^
TPB‐SME	[TPB‐Li]^−^(THF)_1_	−2.83	−1.17	675.2	700.0^[^ ^57^ ^]^
BIP‐SME	[BIP‐Li]^−^(THF)_2_	−2.94	−1.07	643.5	680.0^[^ ^25^ ^]^

## 2 Results and Discussion

PAH‐Li‐SESs represent a class of complex systems formed by dissolving metallic Li in ether‐based solvents containing arenes as additives. These solutions exhibit multifunctional applications, such as serving as anolytes and prelithiation reagents for solid‐state anodes. Prelithiation itself is an advanced technique that addresses irreversible Li loss during initial battery cycles by introducing supplemental Li sources into electrode materials, thereby enhancing both capacity and cycling stability.^[^ ^35^, ^36^, ^51^, ^52^ ^]^

In these systems, Li atoms dissolve in THF solvent, forming solvated Li^+^ cation and e^−^_sol_, collectively denoted as Li‐SESs. This presents a promising chemical prelithiation route. PAHs of specific size ranges are typically introduced as additives to modulate dissolution behavior and enhance cell performance. This enhancement stems from their exceptional structural stability, akin to conventional graphite anode materials. Nevertheless, the precise structural characteristics of PAH‐Li‐SESs remain incompletely understood, and the specific mechanism by which PAHs improve the OCVs of Li‐SES cells warrants further investigation.

To elucidate the structure of Li‐SESs and the functional role of PAHs, we computationally compared the solvation behavior of two Li^+^ ions and two e^−^_sol_ species (formally representing two Li atoms) in THF solvent with and without PAH additives, specifically PAH‐Li‐SESs versus Li_2_@THF systems, using eight distinct PAHs (Figure 1). We establish an AIMD model for each system, comprising two formal Li atoms, 50 THF molecules, and one PAH molecule in a periodic cube box with the side length of 19.20 Å (AN) and 19.29 Å (TER), etc., represented as PAH∙2Li(THF)_50_ (where PAH = AN, TER, etc.). These concentrations (e.g., 0.234 mol L^−1^ for AN or 0.231 mol L^−1^ for TER; Table S1, Supporting Information) closely mirror experimental conditions.^[^ ^25^, ^26^, ^27^, ^28^, ^29^ ^]^

AIMD investigations of three possible electronic states (triplet, closed‐shell singlet, and open‐shell singlet), supplemented by molecular orbital (MO) analyses, confirm the closed‐shell singlet (CS) state as the ground state for all PAH‐Li‐SESs studied (Figures S2–S4, Supporting Information). Consequently, we analyze the CS ground‐state AIMD trajectories to characterize the prevailing binding modes involving Li^+^, e^−^_sol_, PAH molecules, and coordinating THF molecules within the solvent environment, as well as their associated structural dynamics and fluctuations.

### 2.1 Structural Character of Supermolecule Entities

As Li‐SES prelithiation involves dynamic interactions among Li^+^, e^−^_sol_, PAH and THF solvent molecules, understanding their binding modes and configuration fluctuation dynamics is essential for exploring the structural and functional characteristics (e.g., OCVs) of these batteries. To differentiate the role of the PAH additive, AIMD simulations were conducted on two distinct systems: Li_2_@THF (without additives) and PAH‐Li‐SESs, (e.g., PAH = AN, TER).

**Figure 2:**
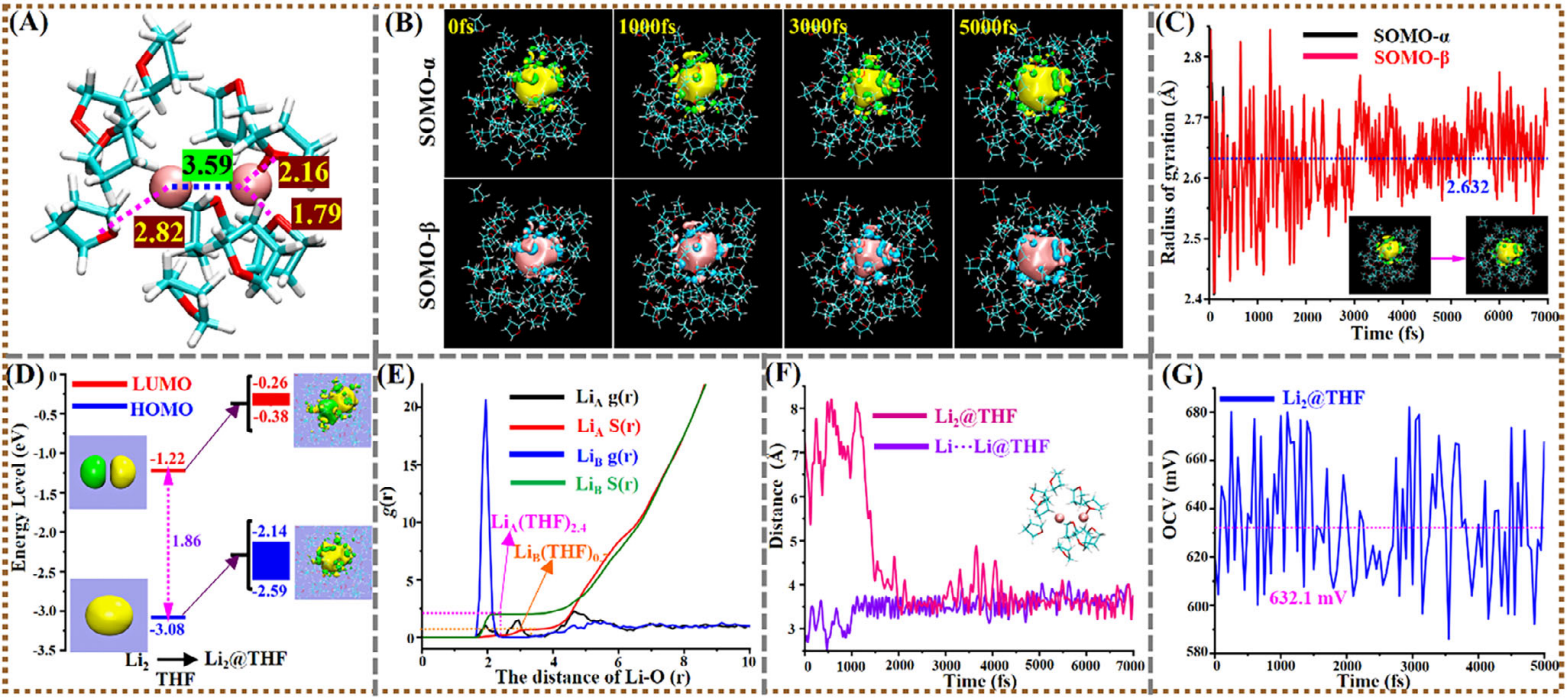
Transient structure and evolution of SME and OCV variation. A) A transient configuration of (THF)_2.4_Li─Li(THF)_0.7_ SME with Li─Li distance and representative Li‐O_THF_ distances (in Å). B) Representative snapshot configurations of Li_2_@THF in its AIMD trajectory and C) time evolution of radii of gyration (Å) of two electrons. D) HOMO/LUMO energies of gaseous Li_2_ and HOMO/LUMO energy bands of Li_2_@THF (i.e., Li_2_‐SME). E) Radial distribution functions (*g*(r)) of Li‐O_THF_ and their integrals for coordination numbers (Li_A_(THF)_2.4_, Li_B_(THF)_0.7_). F) Li∙∙∙Li distance evolutions in two trajectories with different initial configurations both which finally converge to an average distance of ca. 3.59 Å, and G) time evolution of the predicted OCVs for Li_2_@THF.

**(A) Li_2_@THF solution**. AIMD simulations reveal the binding structure and dynamics of two Li atoms in THF (Li_2_@THF), providing details including e^−^_sol_ MO distributions, radii of gyration (*r*_g_) of e^−^_sol_, Li─Li distance and relevant radial distribution function *g*(r) (**Figure** **2**). Results indicate that Li atoms in liquid THF preferentially bind as a solvated Li_2_ molecule, forming an asymmetric (THF)_2.4_Li─Li(THF)_0.7_ SME structure where the THF coordination number per Li is a statistical average (Figure 2). Solvated structures of oligo‐Li clusters (Li_3_@THF and Li_4_@THF) were also examined. AIMD simulations reveal favorable binding modes as Li_3_@THF→Li_2_@THF + Li@THF and Li_4_@THF→Li_2_@THF + Li_2_@THF, respectively, evidenced by Li─Li distances and HOMO distributions (Figures S6–S8, Supporting Information). These results confirm the stability of the Li─Li backbone in the asymmetric (THF)_2.4_Li‐Li(THF)_0.7_ entity despite dynamic ligand exchange and variable THF count.

In this SME structure, 3–4 THF molecules coordinate the Li_2_ dimer, with O─Li distances between 1.80 and 2.80 Å (Figure 2A) and an elongated Li─Li bond (3.59 Å). Coordination imposes Pauli repulsion from the lone‐pair electrons of THF oxygen (O_THF_) atoms, expanding the valence electron (HOMO) distribution of Li_2_. This results in a diffuse and distorted electron distribution characteristic – an electron‐expanded solvated Li_2_ dimer (Figure 2B–D). Analogous entity structures for solvated Na_2_ and K_2_ dimers in THF, albeit with higher coordination numbers,^[^ ^53^, ^54^, ^55^ ^]^ further support this structural motif. The formed SME is thus the exclusive structure for added Li in THF prelithiation solutions, governing electrochemical properties like OCVs (Figure 2G).

The predicted OCV for the Li_2_@THF solution with this SME is ≈632 mV (Figure 2G and Table 1) based on the half‐cell model (Figure 1D). While this solvated Li_2_ SME dictates the OCV of Li‐SESs, analysis reveals two drawbacks: a low OCV and difficulty in further discharging. This limitation stems from the stable discharged structure (Li_2_^+^@THF), which inhibits further electron release, particularly after relaxation (high vertical detachment energy for the second electron, (VDE(2) = 3.547 eV, Table S2, Supporting Information). Additionally, the average HOMO energy (E_HOMO_ = ‐2.14 to ‐2.59 eV; avg. ‐2.38 eV) in the fluctuating Li_2_@THF solution is significantly higher than both the valence band maximum (VBM, −6.54 eV) of THF and the E_HOMO_ (−3.08 eV) of gaseous Li_2_ (Figure 1B,D), indicating destabilization by solvation. Addressing these unfavorable factors is thus necessary to enhance prelithiation efficiency. Incorporating additives like PAHs is a promising strategy, as demonstrated experimentally.^[^ ^25^, ^26^, ^27^, ^28^, ^29^ ^]^

**B) PAH‐Li‐SESs**. While previous studies indicate PAH additives can enhance OCVs,^[^ ^25^, ^26^, ^27^, ^28^, ^29^, ^30^ ^]^ reliable techniques are still lacking, and the underlying mechanisms—such as manipulating Li electrons, modifying entity structures, and optimizing electron‐release for high OCVs—remain unknown.

**Figure 3:**
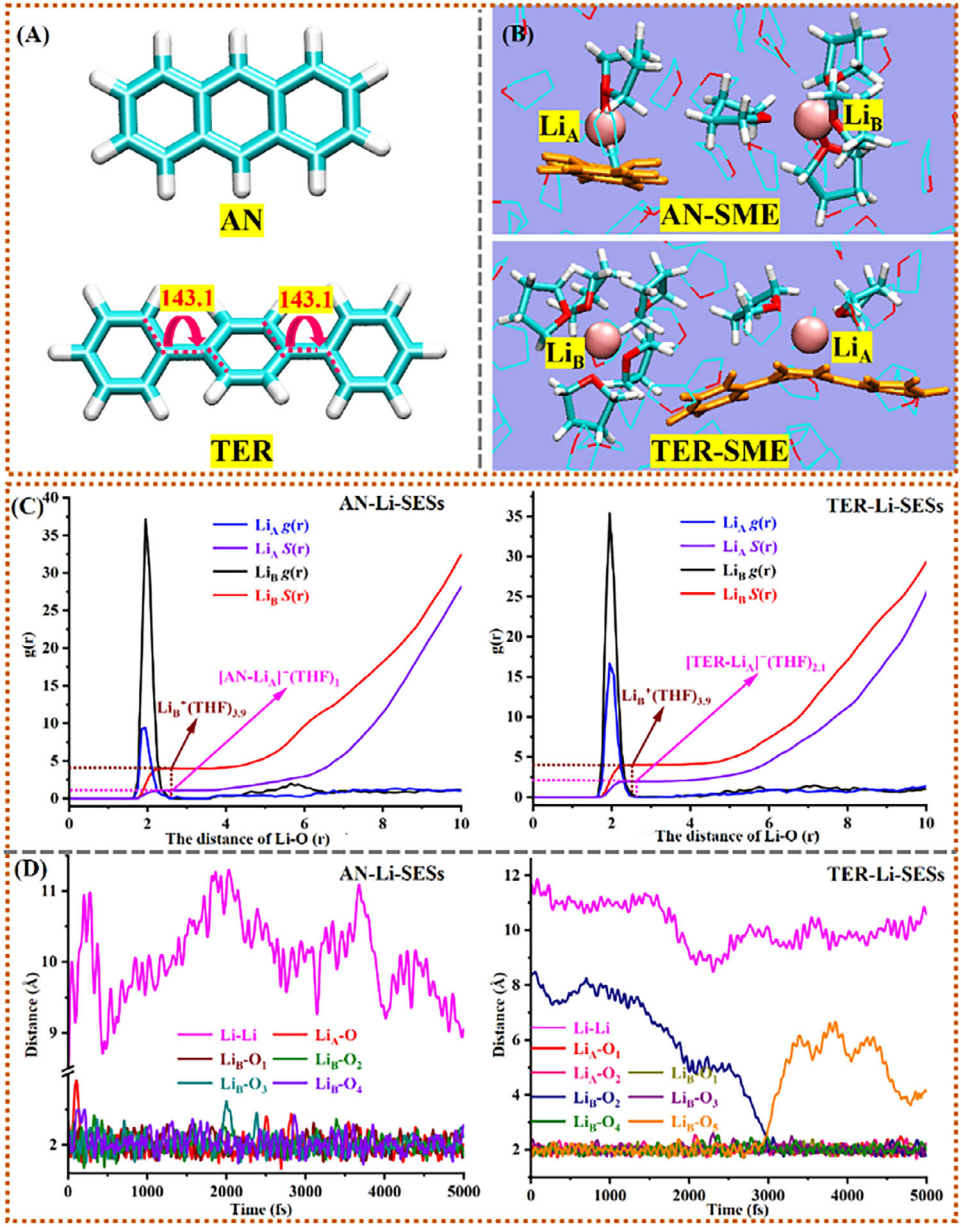
Structures and characterization of AN/TER‐SMEs. A) Molecular structures of AN and TER with two key dihedral angles. B) Structures of trapped electrons of AN‐SME and TER‐SME. C) Radial distribution functions, *g*(r) (black and blue lines), showing the coordinating modes of Li^+^ by O_THF_ for PAH‐Li‐SESs containing [AN‐Li]^−^(THF)_1_ and [TER‐Li]^−^(THF)_2_ with the Li^+^←O_THF_ dative bonds, respectively. D) Time evolutions of Li^+^∙∙∙O distance between Li^+^ and its near THF and Li∙∙∙Li distance for AN‐Li‐SES and TER‐Li‐SES which show the ligand exchange for the latter.

To address this, we examined PAH‐Li‐SESs (PAH = AN, TER, etc., Figure 1). AIMD simulations reveal a distinct structural difference: each system forms an SME and a separate solvated Li^+^ species, unlike Li_2_@THF. In the SME, a PAH traps Li^+^ and e^−^_sol_ assisted by a few THF molecules (**Figure** **3**B; Figure S9, Supporting Information). We characterized local structures by statistically analyzing radial distribution functions (*g*(r)) for Li─Li, Li─O_THF_, and Li‐C_PAH_ over AIMD trajectories (Figure 3C).

For AN‐Li‐SESs, integration of *g*(r) confirms THF coordination numbers of ≈1 for Li_A_ and ≈3–4 for Li_B_. This suggests two moieties: an AN‐SME ([AN‐Li]^−^(THF)_1_) and a Li^+^(THF)_3‐4_ complex (further supported by charge analysis). Similarly, TER‐Li‐SESs form a TER‐SME ([TER‐Li]^−^(THF)_2_) and a Li^+^(THF)_3‐4_ complex. Comparable structures exist for other PAH‐Li‐SESs (Figures S9 and S10, Supporting Information). Compared to (THF)_2.4_Li‐Li(THF)_0.7_ in Li_2_@THF, PAH addition significantly alters Li^+^/e^−^_sol_/THF interactions, yielding SMEs with the common motif [PAH‐Li]^−^(THF)_n_. These entities exhibit distinct electron distributions and energetics relative to the Li_2_@THF SME, contributing differently to OCVs.

Monitoring Li∙∙∙Li (*d*_Li∙∙∙Li_) and Li∙∙∙O_THF_ (*d*_Li∙∙∙O_) distances in representative AN/TER‐Li‐SESs shows *d*_Li∙∙∙Li_ at ≈10 Å (Figure 3D), significantly longer than in Li_2_@THF (3.59 Å). This confirms no interaction between Li species and distinct structural dynamics. Both Li atoms maintain close proximity to coordinating O_THF_ (average *d*_Li∙∙∙O_ ≈ 2.102 Å), indicating strong Li←O_THF_ dative bonds for PAH‐SMEs (PAH = AN and TER). These bonds arise from a balance between valence electron (2Li) Pauli repulsion and Li^+^ core attraction to O_THF_ lone pairs, modulating Li‐electron activity for higher OCVs.

Monitoring PAH‐Li distances (Figure S11, Supporting Information) shows one Li consistently close to a PAH six‐membered ring (average Li∙∙∙C_PAH_ distance: AN‐SME ≈2.427 Å, TER‐SME ≈2.631 Å), while the other remains distant. This indicates strong PAH interaction with only one Li, forming the [PAH‐Li]^−^(THF)_n_ entity; the other Li forms a THF complex.

PAH distortion within PAH‐SMEs was also analyzed. Compared to AN (near‐planar) and TER (twisted) in pure THF (Figure 3A; Figure S12, Supporting Information), AN in AN‐SME exhibits dynamic bending oscillation (≈132°–180°) and TER in TER‐SME shows twisting fluctuation (≈145°–180°). These deformations, driven by Li∙∙∙PAH interaction and THF coordination, disrupt their original conjugation. AN's rigid, fully conjugated planar structure allows extensive π‐electron delocalization. TER's inherent twisting between benzene rings reduces conjugation efficiency. TER's greater structural flexibility leads to more distorted configurations under thermal fluctuation and a larger average deformation energy upon SME formation (TER‐SME: 2.67 eV; AN‐SME: 1.89 eV; Figure S13, Supporting Information). Overall, conjugation weakening due to deformation lowers PAH LUMO energies (E_LUMO_, Table 1; Table S3, Supporting Information), enhancing Li‐electron trapping. Consequently, SME HOMO energies (‐2.56 eV/AN‐SME; ‐2.75 eV/TER‐SME) are lower than ‐2.38 eV observed for (THF)_2.4_Li‐Li(THF)_0.7_.

### 2.2 Electron Distributions and Releasing Ability

Following an analysis of the structural features of these prelithiation entities, it is essential to characterize their electron distributions. The electronic structures of these PAH‐SMEs directly impact both the entities' configurations and the discharge characteristics and mechanisms of PAH‐Li‐SES cells.

**Figure 4:**
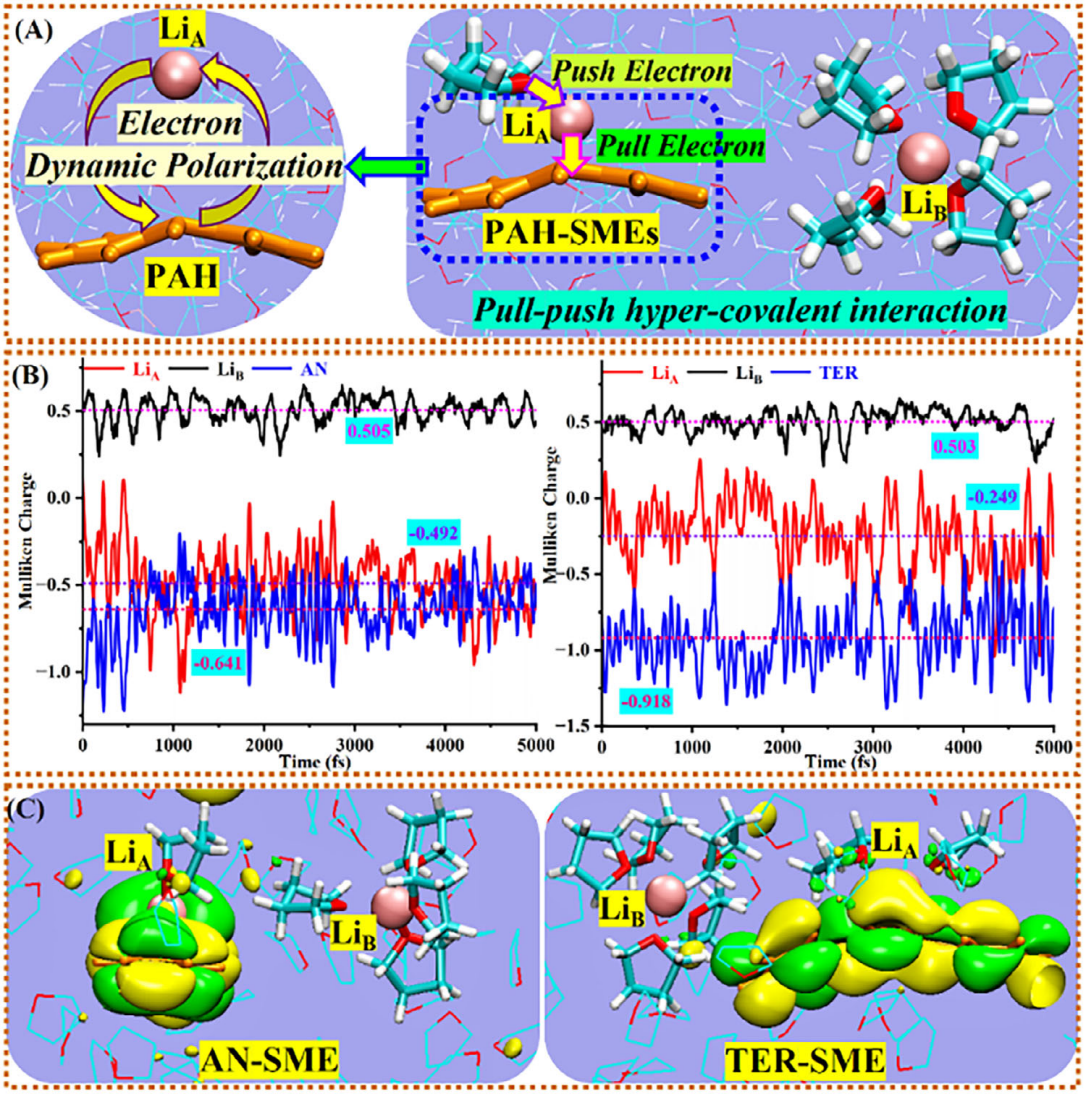
Hyper‐covalent interaction schematic and electron distribution dynamics. A) Structures of two Li‐species and the pull‐push hyper‐covalent interaction and intra‐entity charge oscillation mode. B) Mulliken charge fluctuations of Li_A_, Li_B_ and PAH in AN‐Li‐SES and TER‐Li‐SES during 5 ps AIMD trajectories. Symmetrical oscillations in PAH and Li_A_ charges reveal intra‐entity dynamic polarization. C) Trapped electron distributions in AN‐SME and TER‐SME.

Mulliken charges quantify the electron distributions within the two PAH‐Li‐SESs. In AN‐Li‐SES (**Figure** **4**B), average charges are ‐0.492 (Li_A_), ‐0.641 (AN), and 0.505 (Li_B_), while TER‐Li‐SES shows ‐0.249 (Li_A_), ‐0.918 (TER), and 0.503 (Li_B_). Combined with structural data, the entities are identified as anionic SMEs: [AN‐Li_A_]^−^(THF)_1_ (charge ‐0.996) and [TER‐Li_A_]^−^(THF)_2_ (charge ‐0.953). The spatially separate LiB species exhibit positive charges (0.874 and 0.842, respectively), forming Li_B_^+^(THF)_3‐4_ cation. These distributions suggest electron transfer from coordinating THF ligands to [PAH‐Li]^−^, enabling hyper‐covalent interactions between PAH, Li^+^, and THF. Consequently, PAH‐based SMEs (e.g., AN‐SME, TER‐SME) achieve electron transfer from Li to PAH via THF‐mediated pull‐push hyper‐covalent interactions. In this mechanism, PAH acts as an electron trap while coordinated THFs facilitate electron transfer, synergistically enabling PAH additives to function as prelithiation electron reservoirs.

We monitored electron population dynamics through HOMO evolution (Figure S3, Supporting Information) and Mulliken charge oscillations (Figure 4B) to probe electron stability within PAH‐based SMEs. Despite AIMD simulations using unrestricted singlet states, both Li electrons exhibit identical MO localization on PAH(LUMO). This confirms two opposite‐spin electrons are fully trapped within each SME, comprising PAH, Li^+^ and coordinating THFs. Minimal HOMO shape variation over time confirms high stability of these prelithiation electrons.

Comparisons of transient HOMO distributions (Figure 4C; Figure S14, Supporting Information) further illustrate electron stability. Li_2_@THF SMEs show ellipsoidal electron distributions, while PAH‐Li‐SESs exhibit distorted MO distributions reflecting altered entity structures. PAH additives serve as functional carriers, each trapping two solvated electrons (e^−^_sol_) and one Li^+^, stabilized by coordinating THFs. The non‐PAH‐bound Li^+^ forms distant Li^+^(THF)_3‐4_ complexes, enabling rapid ion transport during discharge. This configuration meets high‐performance cell requirements, with structural stability arising from the synergistic pull‐push effect (Figure 4A). Coordinating THF molecules are thus indispensable for forming [PAH‐Li]^−^(THF)_n_ SMEs and maintaining their structural integrity.

Vertical Detachment Energies (VDEs) quantify the discharge potential of PAH‐SMEs (Table S2, Supporting Information). All PAH‐SMEs exhibit lower first VDEs (VDE(1)) than Li_2_‐SME in Li_2_@THF, indicating comparatively easy discharge. Critically, the second VDEs (VDE(2)*, mono‐cation detachment energies) are 7–30% lower than Li_2_‐SME's 3.547 eV, promoting continuous discharge. This analysis confirms PAH‐SMEs provide robust platforms for balancing stable electron confinement and efficient release.

Solvent Effects. In these PAH‐Li‐SESs, the dual solvent components (THF and PAH) perform multiple functions in manipulating PAH‐entities. Liquid THF serves not only as a dielectric medium dispersing and stabilizing lithium species in both Li_2_@THF and PAH‐Li‐SESs during prelithiation, but crucially, its molecular constituents actively participate in assembling solvated multi‐electron entities (SMEs). Specific THF molecules incorporated within these entities modulate electron distribution and transfer through precisely balanced coordination, repulsion, and polarization effects.

In Li_2_@THF, THF ligands function as steric modulators, confining two e^−^_sol_ within an asymmetric (THF)_2.4_Li‐Li(THF)_0.7_ entity (Figure 2). Their electron densities exhibit defective ellipsoidal distributions due to competing forces: 1) electrostatic Li^+^ attraction pulling e^−^_sol_ toward the dimer core countered by 2) Pauli repulsion from O_THF_ lone‐pair electrons pushing electrons outward into interstitial regions. Critically, THF acts as an inert dopant whose Li^+^∙∙∙O_THF_ coordination polarizes the electron cloud (Figure 2). Molecular orbital analysis confirms no covalent mixing, preserving electron solvation character. This unique solvation activates Li_2_, substantially elongating the Li─Li bond by 0.89 Å (2.70 Å gas phase → 3.59 Å THF), elevating E_HOMO_ by 0.94 eV (‐3.08 eV gas phase → ‐2.14 eV THF), and inducing electron polarization through increased dipole moment (0 Debye gas phase → ≈11 Debye THF). These changes collectively facilitate Li‐electron release during discharge.

PAH additives trigger a solvent‐cosolvent synergy that fundamentally restructures electron distributions in Li‐SESs. The eight studied PAH molecules feature low‐lying LUMOs (E_LUMO_: ‐1.07 to ‐2.85 eV), energetically aligned with Li_2_@THF's VBM (E_HOMO_ = ‐2.14 eV; Figure 2D, Table 1). This resonance enables e^−^_sol_ capture, transforming localized Li_2_ electrons in (THF)_2.4_Li‐Li(THF)_0.7_ into solvated [PAH‐Li]^−^(THF)_1‐2_ SMEs. Within this structure, 1–2 THF molecules mediate electron exchange between Li^+^ and PAH (Figure 4), stabilizing Li^+^ through coordination (including spatially separated Li^+^(THF)_3‐4_). This electron redistribution stems from a synergistic pull‐push effect. Consequently, PAH‐SMEs develop exceptionally large dipole moments (50–95 Debye; Table S4, Supporting Information), vastly exceeding the ≈11 Debye moment of (THF)_2.4_Li‐Li(THF)_0.7_, thereby enhancing stabilization in polar THF. PAHs thus function distinctly from THF—acting as electron acceptors (“pull”) versus THF's repulsive/coordinating role (“push”). Surrounding THF further stabilizes polar entities through dielectric shielding.

The resulting [PAH‐Li]^−^(THF)_n_ entities exhibit stabilized electron distributions and reduced electron‐release resistance during discharge, evidenced by decreased VDEs versus Li_2_@THF (Table S2, Supporting Information). For instance, first VDEs decreased by 0.22 eV for [AN‐Li]^−^(THF)_1_ and 0.08 eV for [TER‐Li]^−^(THF)_2_, while second VDEs dropped by 0.73 and 0.53 eV, respectively, enhancing cell performance, particularly for initial electron release. Overall, these multivalent solvent effects synergistically improve battery efficiency.

THF's role evolves from spatial confinement in Li_2_@THF to polarization enhancement in PAH‐Li‐SESs (Figures 2 and 3). While electronically inert, THF now: 1) amplifies [PAH‐Li]^−^ dipole moments by 3–4 D (Table S4, Supporting Information), 2) lowers electron‐release barriers (reduced VDEs) via inner‐/outer‐sphere solvent synergy (Table S2, Supporting Information), and 3) directs electron flow along PAH‐Li^+^ charge gradients through Li^+^←O_THF_ dative bond vibrations (Figure 4). These cooperative effects manifest as higher OCVs in PAH‐Li‐SESs (712–992 mV) versus Li_2_@THF (632 mV) and enhanced entity stability (observed in undecomposed AIMD trajectories), demonstrating solvent engineering's capacity to tailor electron behavior across molecular to device scales.

The above analyses suggest key design principles: 1) Solvent selection: High‐dielectric solvents with weak coordination (e.g., THF/DME blends) optimize e^−^_sol_ polarization and population without excessive trapping. 2) Additive tuning: PAH cosolvents with E_LUMO_ (‐1.20 to ‐3.0 eV) balance electron affinity and release. 3) Interface control: PAH edge functionalization/internal doping enhances Li^+^ chelation while preserving electron delocalization. Thus, THF's dual roles, steric modulator and polarization amplifier, synergize with PAH's electronic delocalization to establish solvent effects as a multiscale design lever for high‐performance liquid LIBs.

### 2.3 Open‐Circuit Voltage (OCV) and HOMO Energies

OCV represents critical performance parameters for Li‐SES cells and corresponds to the potential difference between positive and negative electrodes under zero‐current conditions.^[^ ^58^ ^]^ Metallic Li serves as the reference electrode for measuring PAH‐Li‐SES OCV in half‐cell configurations.^[^ ^25^, ^26^, ^27^, ^28^, ^29^ ^]^ As Figure 1D illustrates, the half‐cell OCV depends on the electronic chemical potential difference between the Li‐SES anode (µA) and metallic Li cathode (µ_C_), expressed as:

(1) OCV=(μA−μC)/n

where n denotes transferred charge number.^[^ ^59^ ^]^ This Li electronic chemical potential difference can be evaluated from electrode material energy changes at varying Li content,^[^ ^60^, ^61^ ^]^ allowing OCV reformulation as:

(2) OCV=ΦC−ΦA

where *Φ*_C_ and *Φ*_A_ represent cathode and anode work functions, respectively, with *n* = 1 due to single‐electron transfer per Li atom.

Work function (*Φ*) corresponds to the minimum energy required for electron emission from a metallic solid, typically calculated as the Fermi‐vacuum level difference. While polycrystalline Li exhibits *Φ*_C_ = 2.9 eV experimentally,^[^ ^62^ ^]^ first‐principles computation remains challenging since periodic boundary conditions preclude a defined vacuum level.^[^ ^39^, ^40^, ^41^ ^]^ Calculating *Φ* for PAH‐Li‐SES bulk solutions presents particular difficulties. To address this, we introduce a novel model quantifying liquid‐state Li‐SES anodic *Φ*_A_ through SME VDE (Figure 1D), justified experimentally below. Although VDE offers a computationally convenient approximation of the work function, it predominantly captures relative energetic trends across different systems rather than yielding quantitatively accurate absolute values. The observed correlation between VDE and experimental OCVs in this study is likely contingent upon specific system characteristics and may arise from compensating errors. Consequently, the principal utility of VDE in this context lies in enabling rapid comparative screening of PAH candidates, rather than in the precise prediction of operational battery voltages.

**Figure 5:**
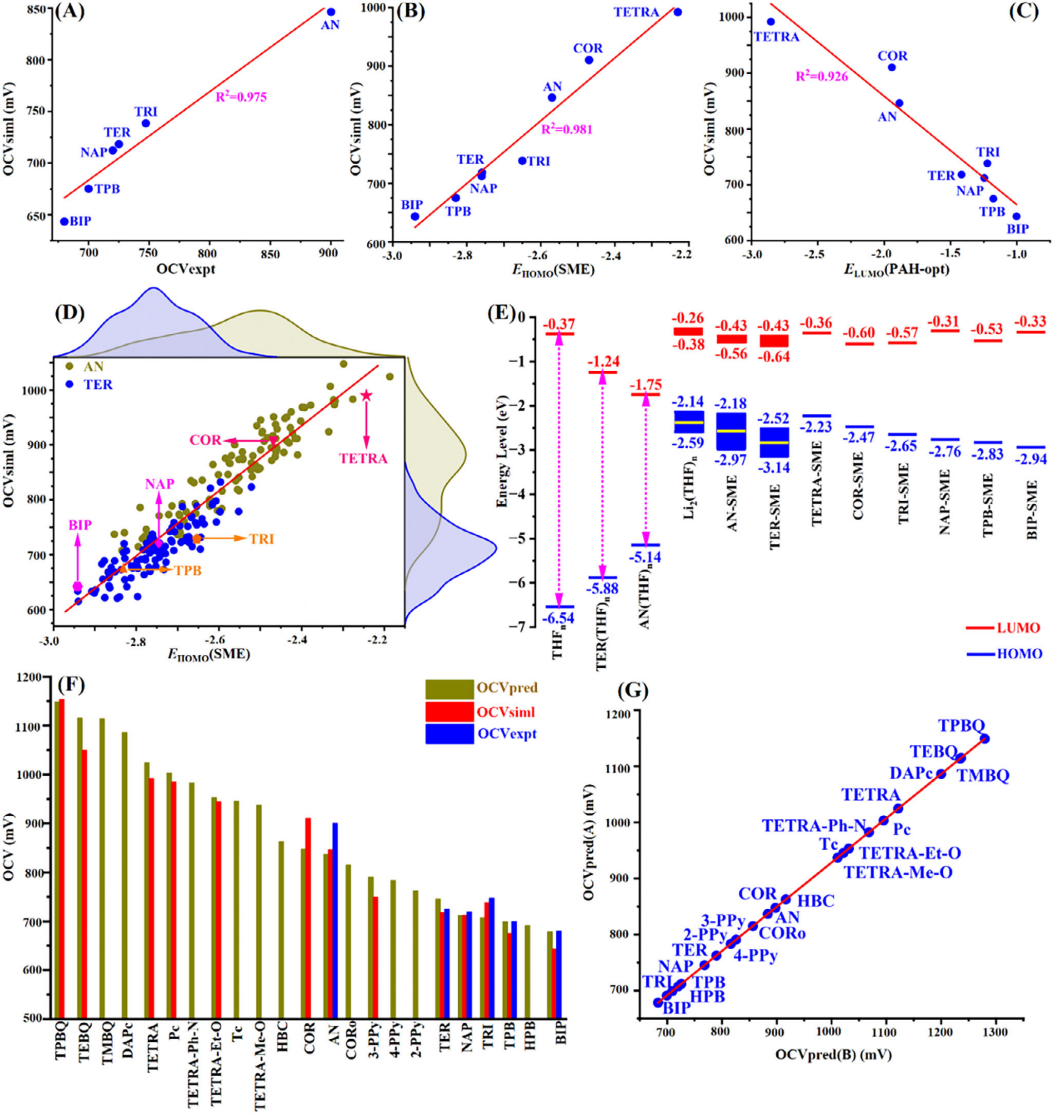
Linear correlations of simulated OCVs (OCV_siml_) with experiments and relevant properties. Linear correlation of OCV_siml_ A) with experimental ones (OCV_expt_) for 6 PAH‐Li‐SESs, and B) with E_HOMO_(SME) for 8 PAH‐Li‐SESs. C) Linear correlations of OCV_siml_ with E_LUMO_(PAH‐opt) of the gaseous structure of PAH. D) Linear configuration of OCV_siml_ using transient configurations with their E_HOMO_(SME) in 200 sampling configurations (100 for each) for two AN/TER‐Li‐SESs. E) E_HOMO_ and E_LUMO_ of solutions for THF solvent (THF_n_), solvated AN (AN(THF)_n_), solvated TER (TER(THF)_n_), and all PAH‐SMEs in THF in which the energy distributions (red/blue energy bands) are displayed for AN‐SME and TER‐SME, and the energy levels of the optimized average configurations are displayed for other PAH‐SMEs. F) Comparison of OCVs among the AIMD‐simulated OCV_siml_, OCV_pred_ predicted by Equation (4) and experimental OCV (OCV_expt_) for all considered PAH additives. G) High linear correlation between OCV_pred_(A) predicted by the expt‐fitting equation (Table S6, Supporting Information) and OCV_pred_(B) predicted by Equation (4), see Table S7 (Supporting Information).

Calculated OCVs for 8 PAH‐Li‐SES systems from dynamics‐equilibrated structures (Table 1) show strong linear correlation with experimental values (**Figure** **5**A). For example, AN/TER‐Li‐SES OCVs (calculated: 846.4 and 718.4 mV; experimental: 900 and 725 mV^[^ ^27^, ^28^ ^]^) demonstrate excellent agreement. Since dissolved Li forms SMEs within PAH hosts, and SME HOMO electrons are discharged, we investigated OCV correlation with equilibrated SME HOMO energies (E_HOMO_(SME)). These exhibit strong linear dependence (R2 = 0.98, Figure 5B), confirming EHOMO(SME) effectively describes Li‐SES OCVs. All PAH‐Li‐SES OCVs (644–992 mV) exceed that of PAH‐free Li_2_@THF solution (633 mV), indicating SME formation elevates OCVs. Elevated E_HOMO_(SME) values (‐2.18 to ‐2.47 eV, Table 1) in AN, COR, and O‐doped TETRA correlate with higher OCVs, though structural dependence is significant.

Solution structural fluctuations dynamically alter SME configurations, influencing electronic properties and OCV stability. Analysis of AIMD trajectories for AN/TER‐Li‐SESs (Figure S15, Supporting Information) reveals Gaussian OCV distributions peaking at 880 and 720 mV, respectively (Figure 5D). The AN‐Li‐SES distribution shows both higher average OCV and broader ranges (500–1050 mV; E_HOMO_(SME): ‐2.20 to ‐3.15 eV) compared to TER‐Li‐SES (550–810 mV; E_HOMO_(SME): ‐2.50 to ‐3.05 eV), indicating greater voltage enhancement but lower stability with AN.

Notably, all transient configurations across both solutions exhibit a robust linear correlation:

(3) OCV=535.78EHOMO(SME)+2200.10

This relationship (Figure 5D) validates E_HOMO_(SME) as a robust OCV descriptor and highlights pull‐push hyper‐covalent interactions in SME as key regulators of cell voltage. Furthermore, since PAHs trap Li electrons via their LUMOs to form the SME HOMO (Figure S18, Supporting Information), PAH electronic properties influence SME energies and OCVs. Strong OCV dependence on PAH E_LUMO_(PAH) emerges (Figure 5C and Table 1; Table S3, Supporting Information):

(4) OCV=−194.17ELUMO(PAH)+470.15

Equations (3) and (4) establish E_HOMO_(SME) and E_LUMO_(PAH) as quantitative descriptors for directly computing OCVs from orbital energies, guiding PAH additive selection to balance Li storage stability with high electron energies for efficient discharge.

Considering solvent‐mediated electron transport during discharge, solution E_LUMO_ remains stable (e.g., AN‐Li‐SES: ‐0.43 to ‐0.56 eV; TER‐Li‐SES: ‐0.43 to ‐0.64 eV) as it represents the THF solvent conduction band minimum (CBM) (Figure S16, Supporting Information). HOMO‐LUMO gaps (ΔE_H‐L_) correlate inversely with E_HOMO_(SME); AN‐Li‐SES exhibits both higher E_HOMO_(SME) (‐2.18 eV) and smaller ΔE_H‐L_ (1.62 eV) than TER‐Li‐SES (EHOMO(SME): ‐2.52 eV; ΔE_H‐L_: 1.88 eV), implying enhanced conductivity.

For liquid‐state LIBs utilizing Li‐SES anodes, optimal PAH selection crucially governs OCV enhancement and reaction mechanism optimization. Dynamic variations (Figure 5D) confirm that AN‐Li‐SES achieves higher OCVs due to greater electron activity (elevated E_HOMO_(SME)), albeit with wider E_HOMO_(SME) distributions arising from larger conformational fluctuations of the AN molecular backbone.

Screening of PAH Additives. Using the established relationship (Equation 4), we predicted the OCVs of 23 PAH‐Li‐SESs (Table S5, Supporting Information), including the previously examined 8 (Table 1) and newly explored 15 compounds. As shown in Figure 5F, the predicted OCVs show strong agreement with experimental values for the six systems where experimental data exists (Table S6, Supporting Information), as well as with AIMD‐simulated values for 13 PAH‐Li‐SESs (Table S5, Supporting Information). Among these 13 simulated systems, 8 PAHs (used to establish the fitting equation) and five newly included PAHs (TPBQ, TEBQ, Pc, TETRA‐Et‐O, and 3‐PPy; Table S7, Supporting Information) were not part of the fitting dataset. This strong correlation establishes E_LUMO_(PAH) as a robust orbital‐based energetic descriptor for additive screening. Predictions indicate benzoquinone‐type derivatives (e.g., TPBQ, TEBQ, TMBQ) possess the highest OCVs (> 1100 mV) among the 23 PAHs evaluated and represent the most promising candidates.

We further validated the OCV prediction method using the experimental fitting equation (OCV_expt_∼E_LUMO_(PAH)) derived from the six experimental PAH‐Li‐SES systems (Table S6, Supporting Information). Predictions generated from both fitting equations (OCV_expt_∼E_LUMO_(PAH) and OCV_siml_∼E_LUMO_(PAH)) exhibit a highly linear correlation (Figure 5G), confirming the reliability of our prediction model (Equation 4) for rationally screening PAH additives in PAH‐Li‐SES anolytes. This consistency further underscores the suitability of PAH LUMO energies as screening descriptors.

## 3 Conclusion

This study combines AIMD simulations and DFT calculations to identify exclusive [PAH‐Li]^−^(THF)_1‐2_ solvated molecular entities (SMEs) as the fundamental voltage‐governing species within Li‐SES anolytes for Li storage and prelithiation. The synergistic aggregation of PAH additives with Li^+^, e^−^_sol_, and THF solvents forms these electroactive chemical entities. PAHs function as π‐conjugated scaffolds that stabilize the charge‐separated states of SMEs via pull‐push hyper‐covalent interactions.

To enable first‐principles calculations of OCVs, we propose an estimation framework for PAH‐Li‐SESs that approximates the work function using VDEs of their constituent SMEs, which provides a practical and reasonably accurate method for linking molecular‐level energetics with experimentally measured voltage values. This approach was validated by the strong agreement between predicted and experimental OCV values across six distinct PAH‐Li‐SESs. Fundamentally, the OCVs of these systems correlate with the electronic properties of their SMEs. We developed a dual‐descriptor predictive framework demonstrating near‐perfect linear correlations between OCVs and SME HOMO energies (spanning 643–992 mV across eight PAH‐Li‐SESs) and PAH LUMO energies. Molecular dynamics reveal that these SMEs exhibit dynamic stability. The dynamic coordination of THF ligands to Li^+^ and π‐electron delocalization within PAHs collectively modulate Li^+^/e^−^_sol_ spatial redistribution and SME E_HOMO_ variations, enabling rational OCV fluctuation.

This work delivers three pivotal advances: 1) First atomic‐resolution identification of SMEs as quantum units governing battery voltage. 2) Predictive VDE/E_HOMO_/E_LUMO_‐OCV relationships for OCV determination, PAH additive screening, and electrolyte design. 3) Molecular engineering principles for manipulating the stability of electroactive Li^+^/e^−^_sol_ species to achieve optimal liquid‐state Li‐ion battery performance.

Our findings establish a quantum‐informed roadmap for liquid Li anodes, yielding solution structures and energetics more favorable than conventional systems. Moreover, the SME concept elucidated here applies not only to Li‐SES batteries but extends to other liquid anodes (e.g., sodium/potassium analogues), providing a new paradigm for next‐generation liquid battery development.

## Conflict of Interest

The authors declare no conflict of interest.

## Supporting information



Supporting Information

## Data Availability

The data that support the findings of this study are available in the supplementary material of this article.
