# Supplementary material for: Metastable π‐Lithium Supermolecule Entities Govern Voltage in Electron‐Rich Electrolytes: Orbital Energetics as Predictive Voltage Descriptors
Source: Adv Sci (Weinh). 2025 Nov 23;13(6):e17037. doi: 10.1002/advs.202517037 (PMC12866869; doi:10.1002/advs.202517037)
Supplement: Supplementary file 1 — Supporting Information [file ADVS-13-e17037-s001.pdf]

Metastable π-Lithium Supermolecule Entities Govern Voltage in Electron-Rich Electrolytes: Orbital Energetics as Predictive Voltage Descriptors

Yiwei Feng, Hui Dong, Xinyu Song, Yuxiang Bu*

School of Chemistry and Chemical Engineering, Shandong University, Jinan, 250100, P. R. China. *Corresponding author: byx@sdu.edu.cn* (Y. B.)

Supporting Information

Contents

1. Details of Computations and Simulations.

1.1 Structure Model of Full-Cell

1.2 Calculational Scheme for Cell OCV in Half-Cell

1.3 AIMD Simulation and Calculation Details

2. Preliminary Examination and Ground State Confirmation for PAH-Li-SESs

2.1 Preliminary Examination for PAH-Li-SESs

2.2 Ground State Confirmation for PAH-Li-SESs

3. Dynamic Characterization of Li_2@THF, Li_3@THF and Li_4@THF in THF Solvent.

4. Detailed Structural Characterization and Relevant Results of PAH and PAH-Li-SESs

5. Direct Predictions of OCVs for Potential PAH-Li-SESs with Unexplored PAH Additive Molecules and Reliability Analyses

6. Additional Trajectories Support the Conclusion

7. References

1. Details of Computations and Simulations

1.1 Structure Model of Full-Cell

As one of the most promising liquid-state active anode materials, Li-SESs is made up of metal Li dissolved in THF solution with additive PAH.[1] The iodine/iodide mixture is dissolved in methanol to form a catholyte solution, which can function as the active material at the cathode.[2] The electrochemical reactions occurring during the discharging and charging of Li-SES//I_2 liquid-state cell at ambient temperature can be illustrated as follows:

$$\text{Anode:} \quad Li^+ + e^- + \Theta \underset{\text{discharge}}{\overset{\text{charge}}{\rightleftharpoons}} \text{Li-SESs} \tag{1}$$

$$\text{Cathode:} \quad 2Li^+ + 2e^- + I_2 \underset{\text{charge}}{\overset{\text{discharge}}{\rightleftharpoons}} 2LiI \tag{2}$$

$$\text{Overall cell reaction:} \quad 2\text{Li-SESs} + I_2 \underset{\text{charge}}{\overset{\text{discharge}}{\rightleftharpoons}} 2LiI + 2\Theta \tag{3}$$

where Θ denotes the PAH/THF solution.[2-3] In both the charged and uncharged cases, LiI functions as the supporting electrolyte in both anolyte and catholyte. During the charging process, the interaction among Li^+, electrons, THF and PAH cosolvent on the electrode side of Li-SESs results in the formation of Li-SESs. When the cell is discharged, the Li-SESs electrode side dissociates Li^+ and electrons. The aim here is to elucidate the binding modes between the solutes (Li atoms) and solvents (THF and PAH) within Li-SESs anolyte when the liquid-state cell is fully charged but has not yet started discharging. To accurately reinstate the authentic condition within Li-SESs anolyte and obtain Li^+ ions and electrons in liquid THF, 5 ns classical molecular dynamics simulation and 2 ps AIMD simulations of the Li atom plus PAH molecule in liquid THF under periodic boundary condition were conducted employing the Materials Studio/Forcite package [4] and CP2K/Quickstep [5] software package, respectively. Furthermore, the structures for various possible binding modes between Li atoms and PAH with PCM model for solvent effect were optimized with frequency calculations for confirming the binding modes using the Gaussian 16 program.[6]

1.2 Calculational Scheme for Cell OCV in Half-Cell

OCV of a liquid-state Li-SESs cell is the chemical potential difference between the positive electrode and the negative electrode when no current flows through the cell.[7] In

general, the metal Li is employed as reference electrode to measure OCV of Li-SESs cell on the basis of PAH in half-cell experiment.[1d-1e] The electrochemical reactions of Li//Li-SESs liquid-state half-cell can be illustrated as follows:

Oxidation half-reaction: Li-SESs → Li^{+} + e^{-} + Θ (4)

Reduction half-reaction: Li^{+} + e^{-} → Li (5)

Overall reaction: Li-SESs → Li + Θ (6)

where Θ denotes the PAH/solvent solution.[3] As shown in Figure 1D, OCV is determined in accordance with the chemical potential difference of electrode between Li-SESs anode (μ_A) and metal Li reference electrode (i.e., cathode, μ_C) in the half-cell.[8] The relevant formulas can be expressed as

$$\mathrm{OCV} = \frac{\mu_A - \mu_C}{ne} \tag{7}$$

where n is the number of the charge transferred in the cell reaction.[8] Further, Ceder, *et al.* demonstrated that the difference of Li chemical potential can be directly evaluated by the energy change of electrode materials with different Li content.[9] Therefore, on the basis of the above studies, the cell OCV could be written by

$$\mathrm{OCV} = \frac{\Phi_C - \Phi_A}{ne} \tag{8}$$

where Φ_C and Φ_A are the cathode and anode work functions, respectively, and n is set to be 1 because Li loses one electron during cell reaction. Φ is the minimum energy required to kick an electron out of the interior of a metallic solid and is usually estimated as the energy difference between the Fermi level and vacuum level. Although the experimental work function of polycrystalline Li (Φ_C) metal is 2.9 eV,[10] such a definition makes it challenging to calculate the Φ of a solid from first-principles because the vacuum level is very difficult to determine due to the nonexistence of a real vacuum region within a three-dimensional periodic system.[11-14] In particular, for the present PAH-Li-SES systems, it is a challenging task to develop an effective way so that the Φ of such bulk solutions can be calculated. Here, we propose a novel model to effectively characterize Φ_A of the liquid-state Li-SESs anode from first principles (Figure 1D). That is, Φ_A is derived from vertical detachment energy (VDE) of SME, which is suitable for characterizing the electron-loss nature for the Li-SESs anode.

1.3 AIMD Simulation and Calculation Details

AIMD simulation is a powerful tool for elucidating the dynamics and mechanisms for the complex reaction systems, and it enables the identification of intermediates and active participants on the reaction proceeding, essentially providing a statistical population of the states during the reaction. On this basis, the AIMD simulations were performed on the studied systems. Periodic boundary conditions were applied in the calculations for AIMD simulations. The system consists of two Li^+ ions, two electrons, and 50 THF molecules with (PAH-Li-SESs) and without (Li_2@THF) one PAH additive and a series of PAHs are considered such as AN, TER and others (Figure 1) in a periodic cubic box with a fixed side length. PAH additives were selected based on their different structural characteristics, including molecular size, π-conjugation length and experimental evidence.[1] The modelling system for AIMD simulations corresponding to a solution concentration of 0.234 mol/L for AN and 0.231 mol/L for TER, etc. (Table S1). The simulation was implemented in the NVT ensemble utilizing a time-step of 1 fs with a canonical sampling using the Nosé-Hoover thermostat.[15] The Perdew-Burke-Ernzerhof (PBE) exchange correlation functional in combination with the empirical dispersion correction [16] was adopted, which has been widely applied to predict the properties of solutions reasonably. The Goedecker-Teter-Hutter pseudopotentials (GTH) [17] for the core electrons together with a hybrid Gaussian and plane wave (GPW) [18] scheme was used for evaluating efficiently the forces and energies. On the other hand, the self-consistent field calculations were accomplished with a convergence criterion of 10^{-6} Hartree on the total energy in this work. The temperature was carefully maintained at 295.15 K (an appropriate experimental temperature for Li-SESs) to assure a frank diffusive motion.[1f-1g] Moreover, the Kohn-Sham orbitals were expanded into the DZVP basis sets [19] for H, Li, C, and O, and the valence electrons were expanded into plane waves with a cutoff of 300 Ry. The B3LYP (Becke 3 parameter exchange, Lee-Yang-Parr correlation) hybrid functional [20] in combination with a 6-311++G(d,p) basis set was used to obtain a basic understanding of molecular characters for various [Li^+, e^-, PAH] complexes together with possible THF molecules in THF solution.

Table S0. Arrangement for All Considered PAH Systems. (**A**) Predicting OCV_{pred} using the expt-fitting equation, while (**B**) predicting OCV_{pred} using the siml-fitting equation.

No	PAHs	OCV_{expt}	Fitting equation	OCV_{siml}	Fitting equation	OCV_{pred} (A)	OCV_{pred} (B)
1	TPBQ			√		√	√
2	TEBQ			√		√	√
3	TMBQ					√	√
4	DAPc					√	√
5	TETRA-Ph-O			√	√	√	√
6	Pc			√		√	√
7	TETRA-Ph-N					√	√
8	TETRA-Et-O			√		√	√
9	Tc					√	√
10	TETRA-Me-O					√	√
11	HBC					√	√
12	COR			√	√	√	√
13	AN	√	√	√	√	√	√
14	CORo					√	√
15	3-PPy			√		√	√
16	4-PPy					√	√
17	2-PPy					√	√
18	TER	√	√	√	√	√	√
19	NAP	√	√	√	√	√	√
20	TRI	√	√	√	√	√	√
21	TPB	√	√	√	√	√	√
22	HPB					√	√
23	BIP	√	√	√	√	√	√

Table S1. Representative additive PAH structures and their HOMO and LUMO (isosurface = 0.01) in the gas phase, calculated at the B3LYP/6-311++G(d,p) level of theory, and the corresponding solution concentrations (mol/L) and box side length (Å) of the studied model systems for AIMD simulations.

Additive Molecules	Structures	HOMOs	LUMOs	Concentrations of PAH	Box side length
AN	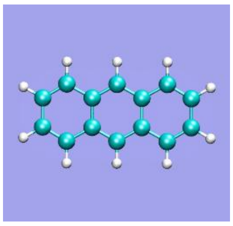	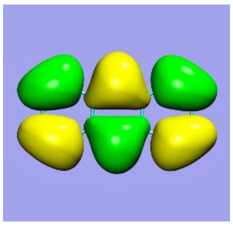	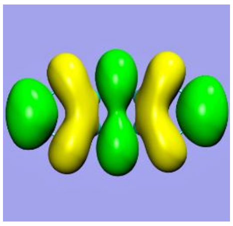	0.234	19.20
TER	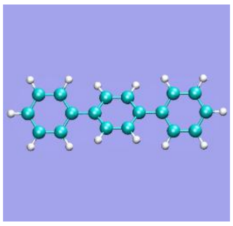	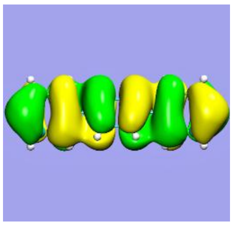	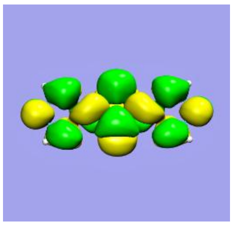	0.231	19.29
NAP	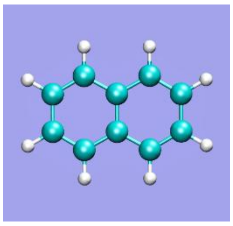	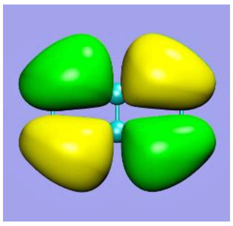	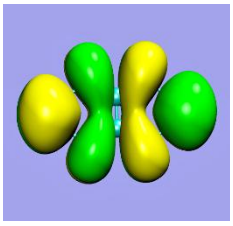	0.237	19.12
BIP	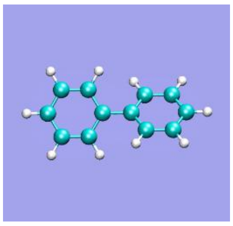	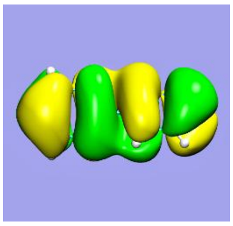	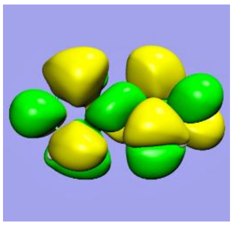	0.236	19.16
TRI	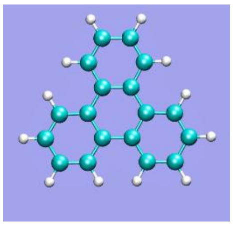	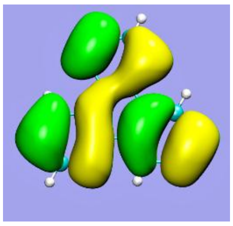	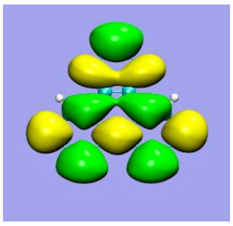	0.227	19.40

TPB	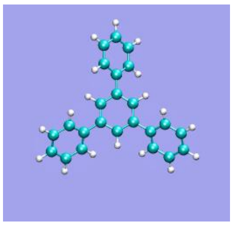	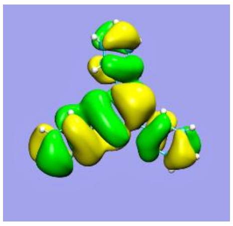	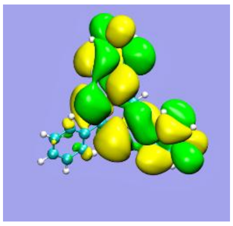	0.223	19.50
COR	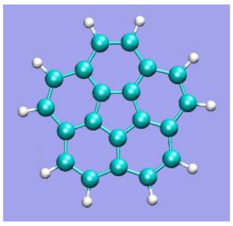	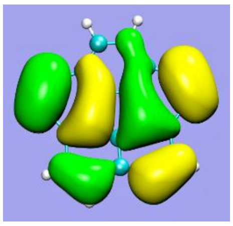	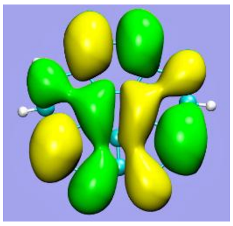	0.230	19.30
TETRA	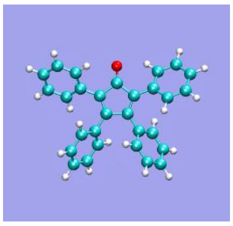	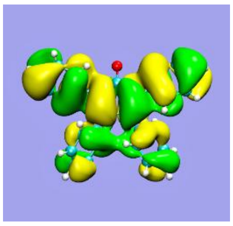	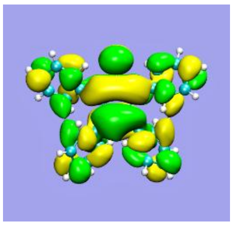	0.223	19.50

2. Preliminary Examination and Ground State Confirmation for PAH-Li-SESs

2.1 Preliminary Examination for PAH-Li-SESs

To confirm the role of PAH as a stabilizer for both Li^+ and electrons in the THF solution, we firstly used DFT method to calculate the relative energies of different PAH-2Li$(THF)_2$ (PAH=AN and TER) complexes using the PCM scheme. The calculations were performed on geometry-optimized configurations from the initial guess, considering various scenarios where Li atoms are positioned either on the same or different sides of PAH (AN and TER). The energies of the CS and T states were initially calculated for four binding modes, and a comparison was made to determine their relative stability (Figure S1). The calculated results indicate that these optimized configurations display the CS ground state, with the structures of 1,2-AN binding mode and 1,5-TER binding mode which exhibit the lowest energy and the highest stability, respectively. In fact, the binding of PAH and Li^+/e^-_{sol} contact pair in solvent are influenced not only by their inherent molecular vibrations but also by transient impact arising from solvent molecules, including direct interaction of the inner-sphere solvent

molecules and dielectric effect of the outer-sphere surrounding solvents. The resulting transient structural distortion and solvent impact can modify the binding modes between PAH and Li^+/e^-_{sol} contact pair and their electronic properties by modulating molecular orbital characters (including energies and spatial distributions). Thus, it is necessary to examine the solvated structural characteristics of the binding modes between PAH and Li^+/e^-_{sol} contact pair.

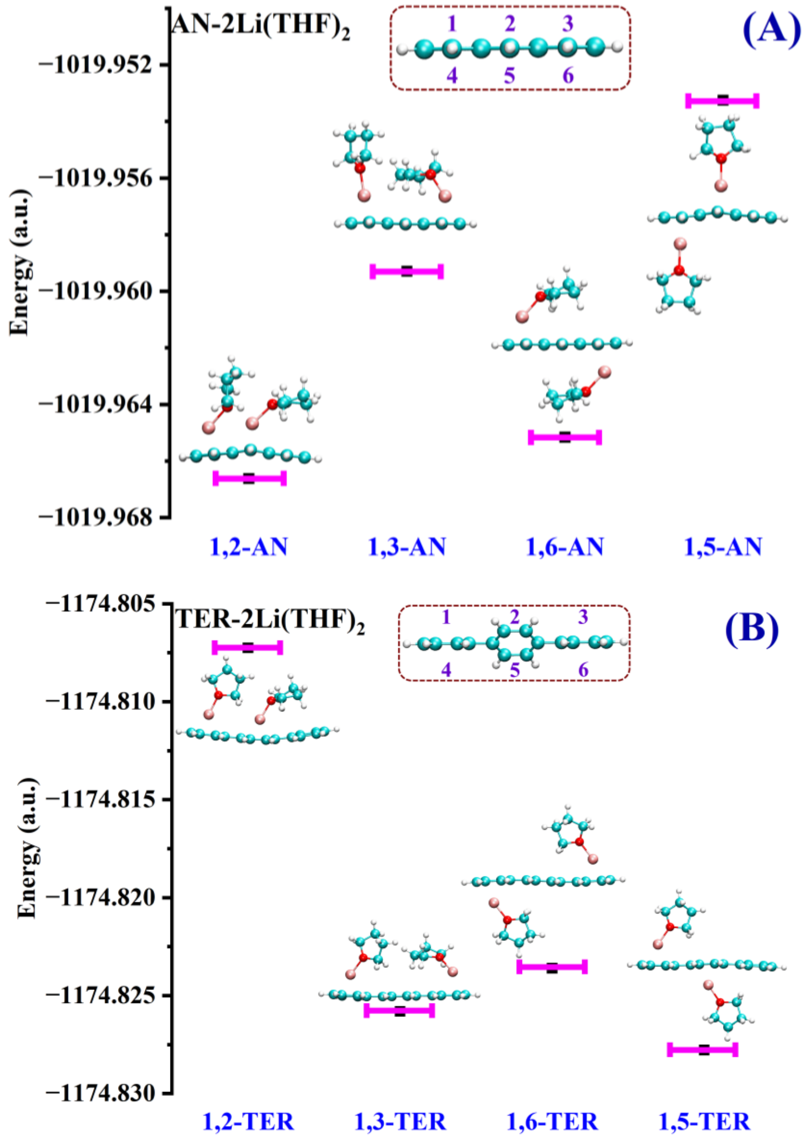

Figure S1. Structural configurations and relative energies for four different binding modes between two Li atom and PAH (AN and TER). Insert pictures are the schematic structures of AN and TER with top view.

2.2 Ground State Confirmation for PAH-Li-SESs

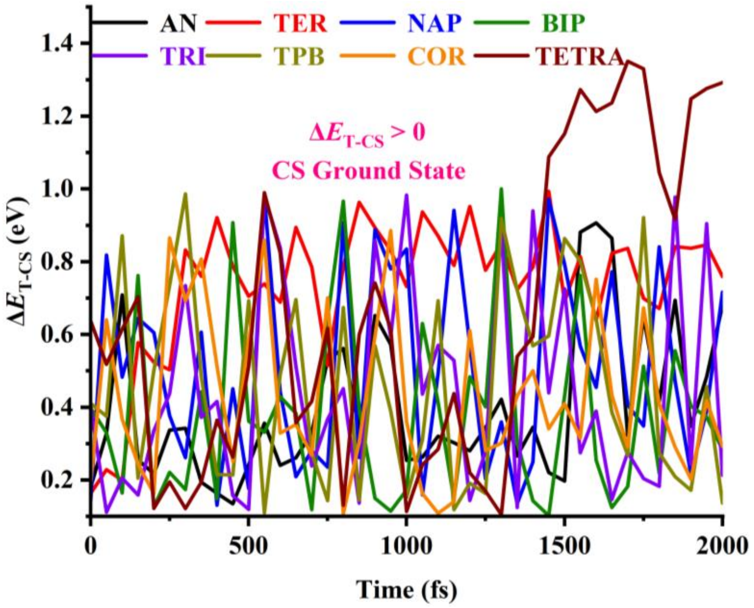

Figure S2. The energy gap $\Delta E_{T\text{-}CS}$ ($\Delta E_{T\text{-}BS} = E_{triplet} - E_{singlet}$) between the closed-shell singlet (CS) and triplet (T) state for PAH-Li-SESs containing different PAH (PAH=AN, TER, NAP, BIP, TRI, TPB, COR or TETRA, Table S1) in their *ab initio* molecular dynamics trajectories, showing that the CS state is the ground state for PAH-Li-SESs as evidenced by $\Delta E_{T\text{-}CS} > 0$.

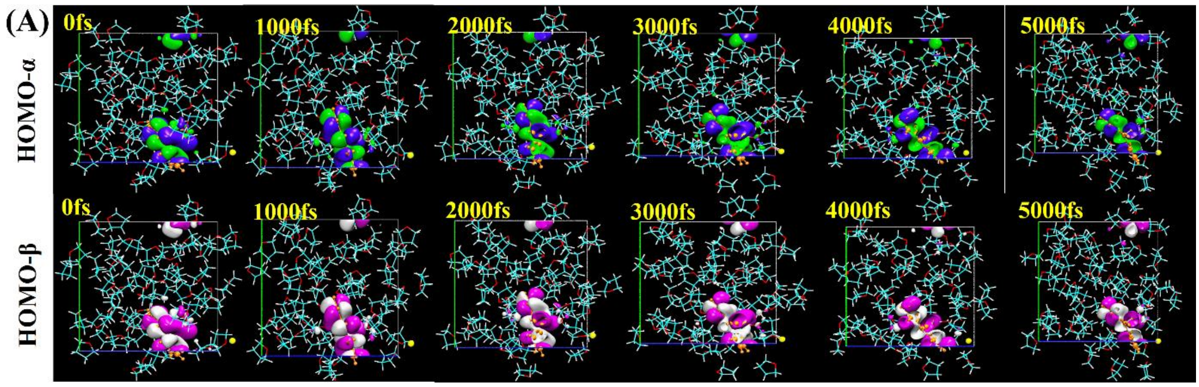

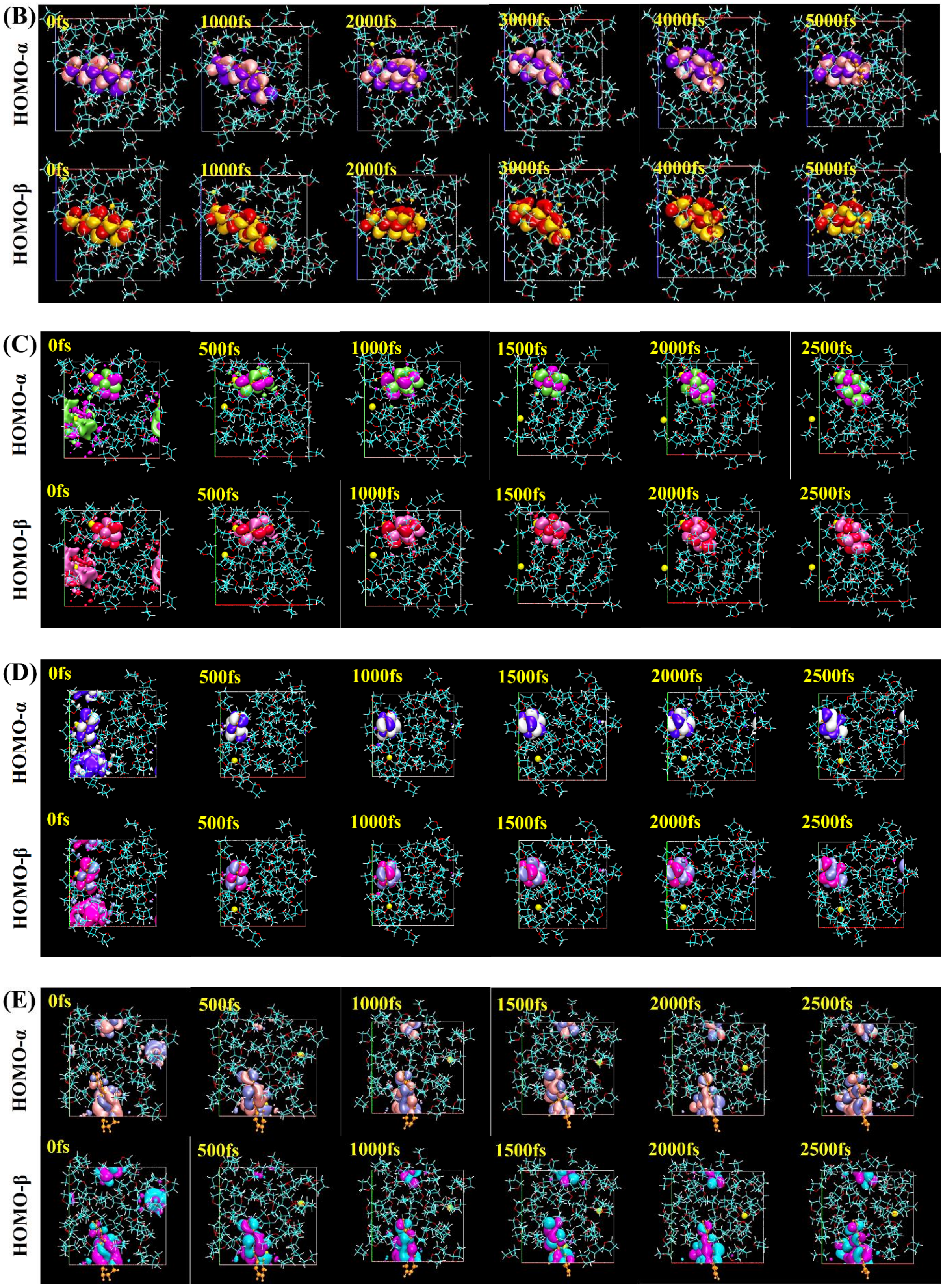

(B)
HOMO-α
HOMO-β
0fs
1000fs
2000fs
3000fs
4000fs
5000fs
(C)
HOMO-α
HOMO-β
0fs
500fs
1000fs
1500fs
2000fs
2500fs
(D)
HOMO-α
HOMO-β
0fs
500fs
1000fs
1500fs
2000fs
2500fs
(E)
HOMO-α
HOMO-β
0fs
500fs
1000fs
1500fs
2000fs
2500fs

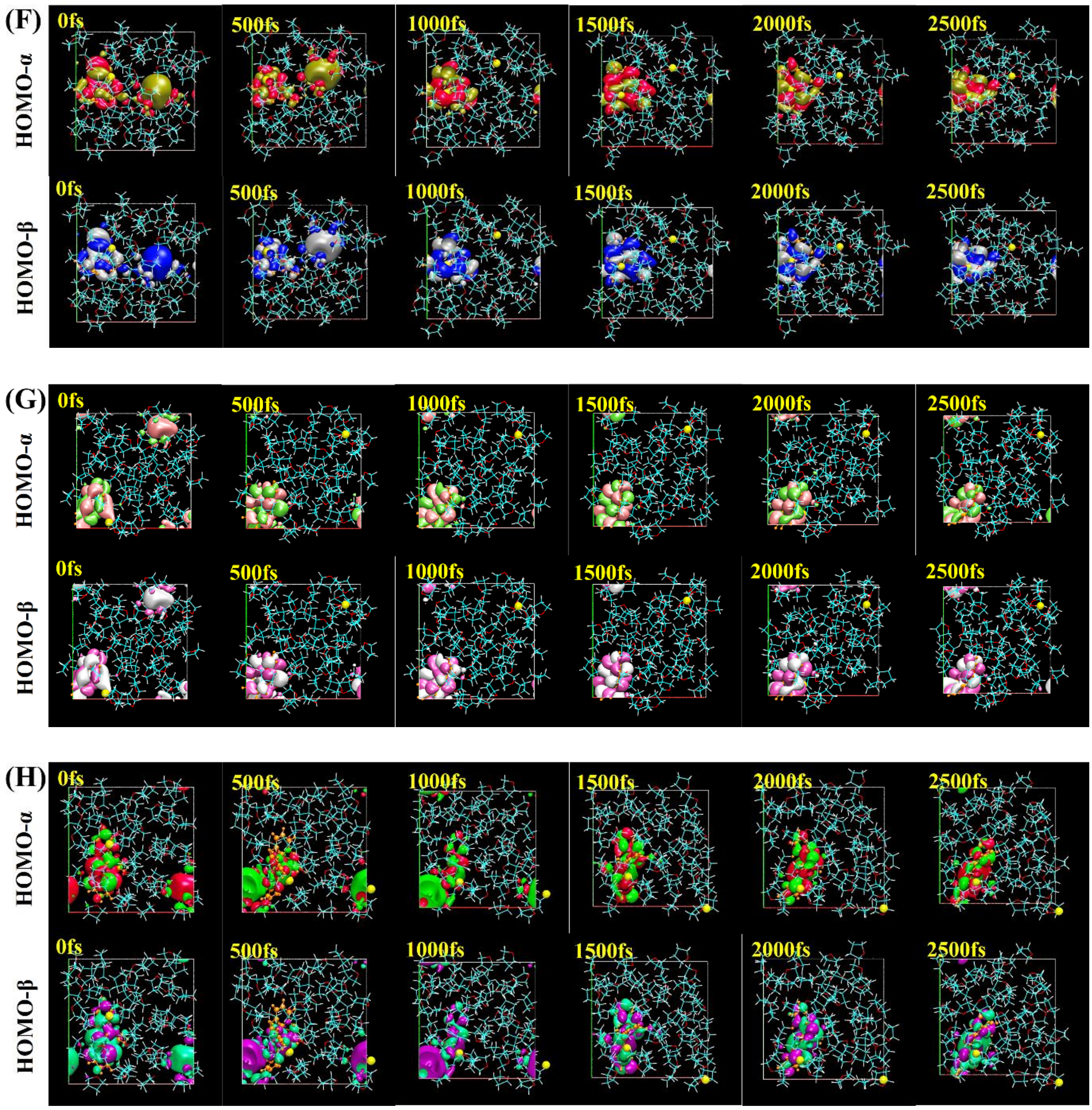

Figure S3. HOMO-α and HOMO-β distributions (isovalue = 0.01) of some representative snapshot at different times extracted from the dynamic trajectories of Li-SESs containing different PAH, including AN (A), TER (B), NAP (C), BIP (D), TRI (E), TPB (F), COR (G) and TETRA (H). The distribution of HOMO-α and HOMO-β remains constant for Li-SESs containing PAH molecules, providing evidence for the ground state being in a CS state.

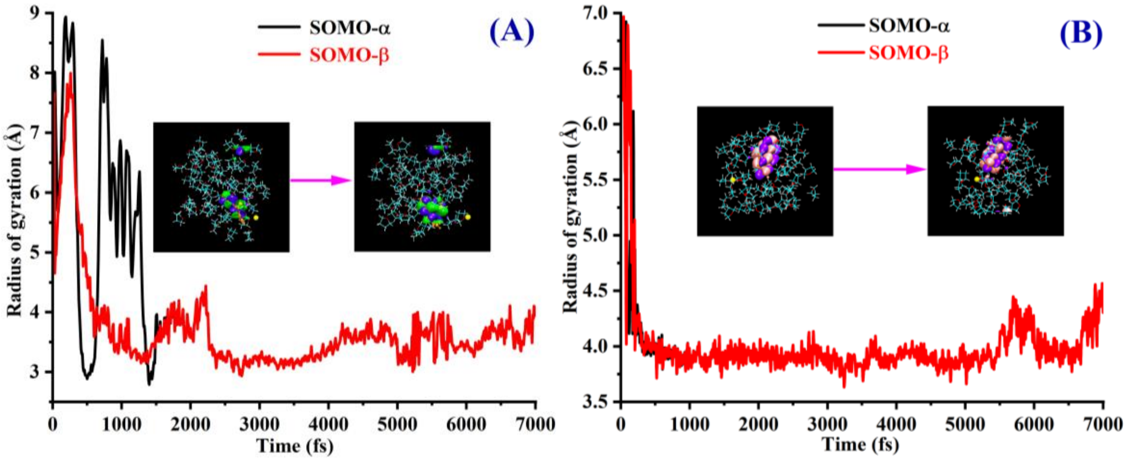

Figure S4. Variation in the radius of gyration (r_g) for α-electron and β-electron with time evolutions in the Li-SESs with AN (A) and TER (B) trajectories. The insert shows the representative HOMO distributions (isovalue = 0.01) of electron distributions at 0 ps and 5 ps for the Li-SESs systems with AN and TER, respectively.

3. Dynamics Characterization of Li_2@THF, Li_3@THF and Li_4@THF in THF Solvent

The behavior of two Li atoms (Li^+/e^-_{sol}) in THF solution was investigated through two different simulation models: one possibility is that two Li atoms exist as a dimer in THF solution, while the other possibility is that they exist independently. Two distinct electronic spin states were considered for the two electrons: T state and BS singlet. The singlet-triplet energy gap ($\Delta E_{T\text{-}BS} = E_T - E_{BS}$) was calculated, and the results featuring all $\Delta E_{T\text{-}BS} > 0$ (Figure S5) suggest that BS state is more stable than T state. The SOMO-α and SOMO-β orbitals in BS state display identical orbital distributions, confirming the fact that CS state is ground state for Li_2@THF system **(**Figure 2B**)**. The radial distribution functions (RDF, g(r)), the radius of gyration (r_g) for electrons and OCVs for Li_2@THF system are investigated (Figure 2C, 2E and 2G). Moreover, the distance of Li···Li for two different simulations show that two Li atoms exist in the form of dimers **(**Figure 2F). Given the widespread recognition of the radial distribution function (RDF, g(r)) as a highly effective and significant approach for reflecting local interactions between alkali metal and THF solvent, we firstly characterize the local interactions between Li^+ and THF by examining the RDF of Li-THF(O) for Li_2@THF system. As shown in Figure 2E, a large and sharp peak is observed in $g(r)$ and the peak position appears

at ca. 1.95 Å for the Li_2@THF dynamics trajectories, indicating the dative-bonding interaction of THF(O) sites and Li^+ core. A continuous coordination number defined by counting the number of THF(O) that distribute within a special distance of the center of each Li^+ core is employed to analyze the coordination number on each Li^+ core in THF. By utilizing the integral of the first trough describing the radius of the first solvation shell and the maximum of Li-THF(O) coordination distance for $g(r)$, we compute the number of coordinating THFs on each Li^+ core. Integration of the $g(r)$ peak for the Li_A and Li_B presents an average of 2.4 and 0.7 THF-O atoms which form dative bond to each Li^+ core, respectively. Furthermore, three Li atoms and four Li atoms in THF solution (i.e., Li_3@THF system and Li_4@THF system) were also investigated by AIMD simulations, respectively. The results indicate that Li atoms exhibit a propensity to exist in dimeric form when dissolved in THF solvent. For the Li_3@THF system, the presence of the spatially separated Li_2···Li species is observed in THF solvent. For the Li_4@THF system, the formation of the separated Li_2···Li_2 complex can be detected in THF solvent. The representative snapshot configurations are shown below.

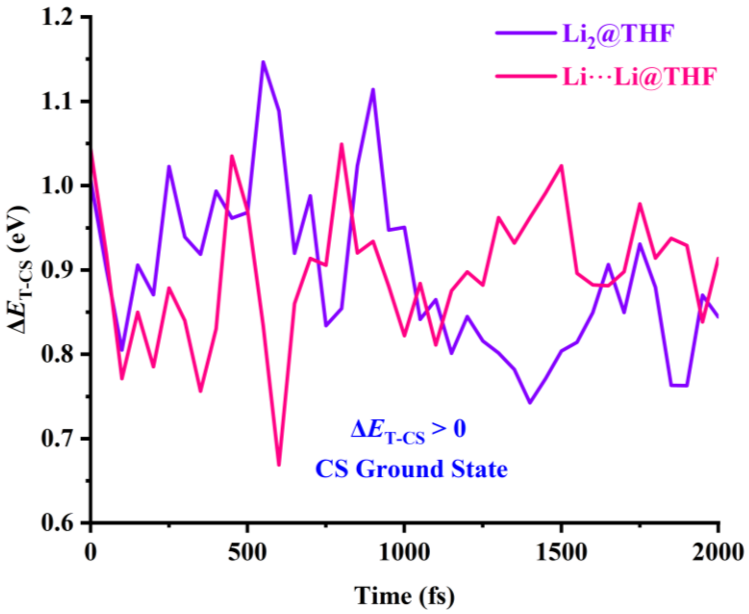

Figure S5. Time evolutions of energy gaps between the CS and T state ($\Delta E_{\text{T-BS}}$) for Li_2@THF (violet line) and for Li···Li@THF (pink line) in THF solvent, respectively, displaying the CS state is their ground states when two Li atoms exists in THF solution. The difference between two curves (Li_2@THF vs Li···Li@THF) is the AIMD simulations using different initial configurations for AIMD simulations.

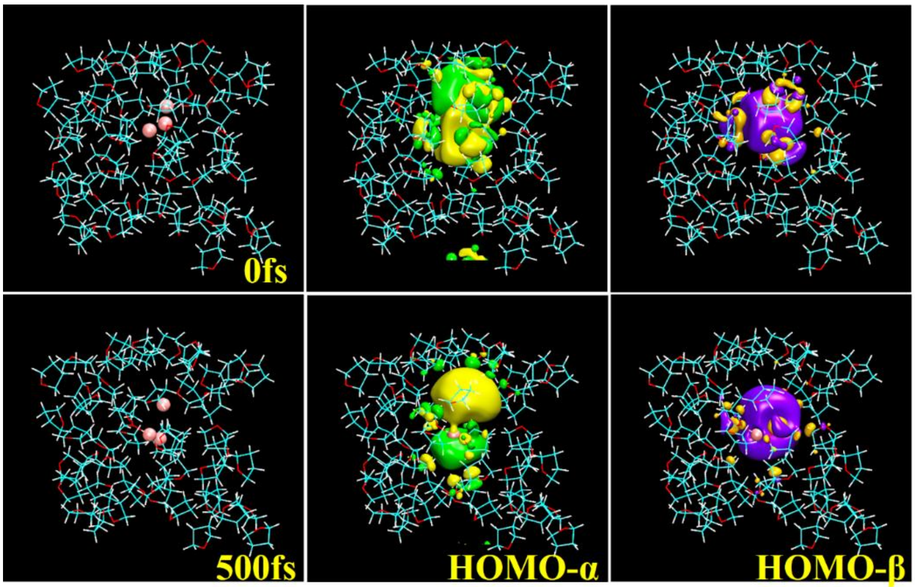

Figure S6. Time evolution of structure, HOMO-α and HOMO-β distributions for Li_3 in THF solution, indicating that the formation of Li_3@THF→ Li_2@THF + Li@THF.

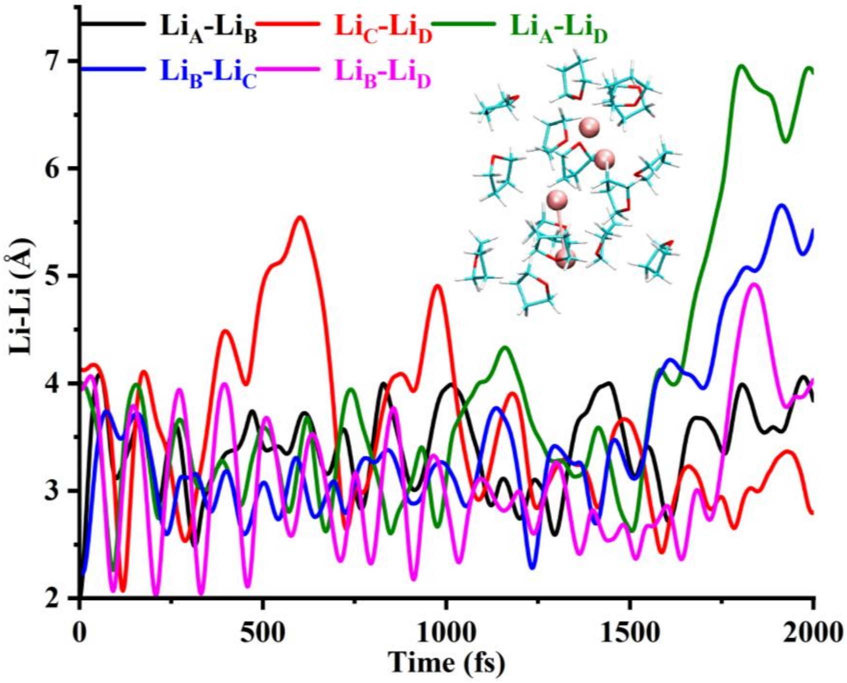

Figure S7. Variations in distances of $d_{Li...Li}$ in the 2 ps AIMD trajectory for Li_4@THF solution, displaying the formation of the $(THF)_xLi_2\cdots Li_2(THF)_y$ mode.

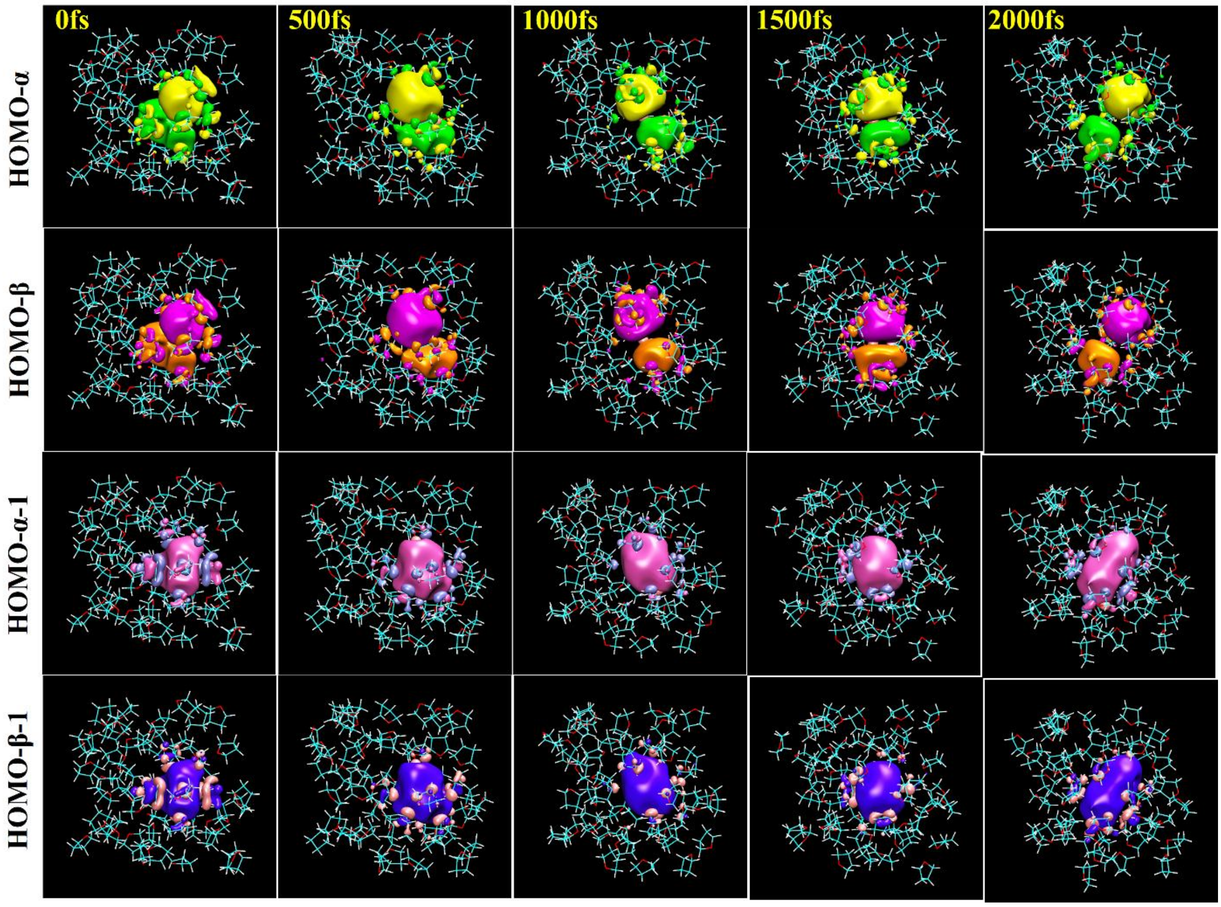

Figure S8. Time evolutions of HOMO-α, HOMO-β, HOMO-α-1, HOMO-β-1 distributions (isovalue = 0.01) of some representative snapshot for Li_4@THF in THF solution. The distribution of SOMO-α and SOMO-β remains constant, providing evidence for the ground state being in a CS state of Li_4@THF system and the Li_4@THF changing into Li_2@THF + Li_2@THF.

4. Detailed Structural Characterization and Relevant Results of PAH and PAH-Li-SESs

Table S2. First/Second Vertical Detachment Energies (VDE(1), VDE(2)*, eV) of the Supermolecule Entities (SMEs). VDE(2)* are calculated using the optimized configurations of the corresponding +1 cations of these entities which reflect their subsequent discharging ability of the entities after losing an electron.

SMEs	VDE(1)/eV	VDE(2)*/eV	ΔVDE/eV
$(THF)_{2.4}Li\text{-}Li(THF)_{0.7}$	2.268	3.547	1.279
$[TETRA\text{-}Li_A]^{-}(THF)_1$	1.908	2.504	0.596
$[COR\text{-}Li_A]^{-}(THF)_2$	1.990	2.576	0.586
$[AN\text{-}Li]^{-}(THF)_1$	2.054	2.815	0.761
$[TRI\text{-}Li_A]^{-}(THF)_1$	2.161	2.976	0.815
$[TER\text{-}Li]^{-}(THF)_2$	2.182	3.019	0.837
$[NAP\text{-}Li_A]^{-}(THF)_2$	2.188	3.198	1.010
$[TPB\text{-}Li_A]^{-}(THF)_1$	2.225	3.291	1.066
$[BIP\text{-}Li_A]^{-}(THF)_2$	2.257	3.312	1.055

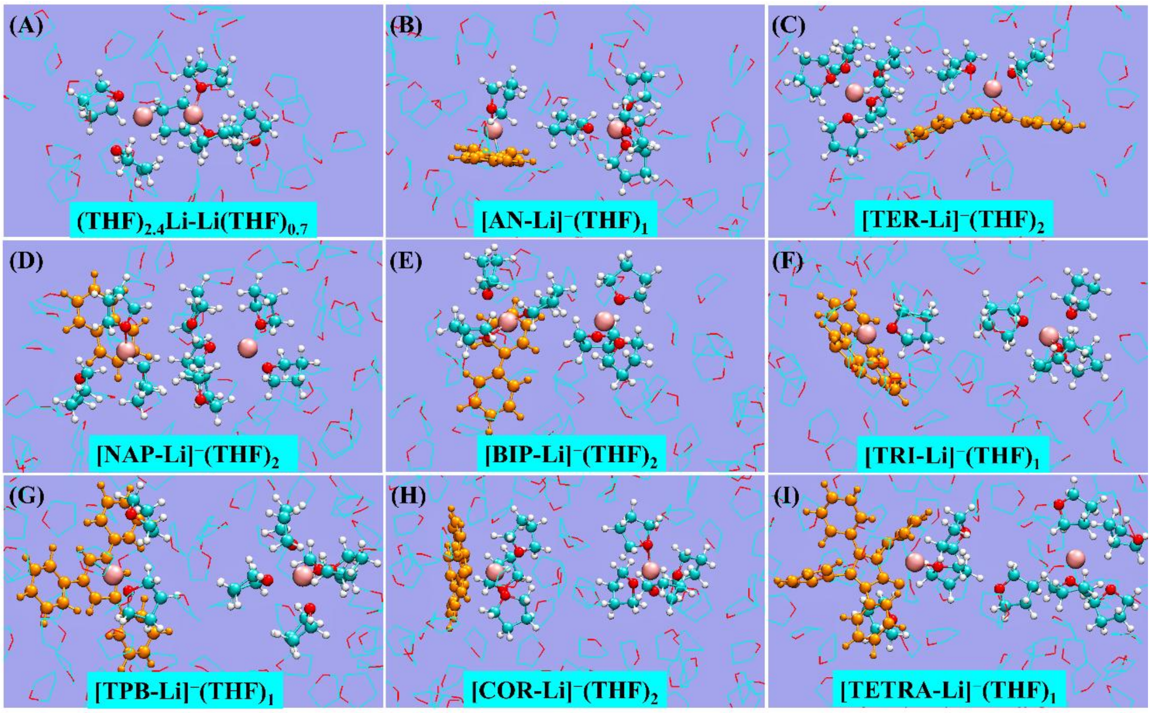

Figure S9. Representative AIMD snapshots of Li_2@THF **(A)**, PAH-Li-SESs (PAH=AN, TER, NAP, BIP, TRI, TPB, COR and TETRA) **(B-I)** showing the formation of novel SMEs in Li-SESs. The proximate surrounding THF molecules are plotted as bright turquoise sticks with red oxygen atoms. PAHs are displayed with bright orange backbone. The solvated Li^+, together with its coordinating THF molecules, is also presented. The 2 ps transient snapshot structures for $(THF)_{2.4}$Li-Li$(THF)_{0.7}$ for Li_2@THF and [PAH-Li]$^{-}$$(THF)_n$ (n=1-2) for PAH-Li-SESs are extracted from the corresponding AIMD trajectories, respectively.

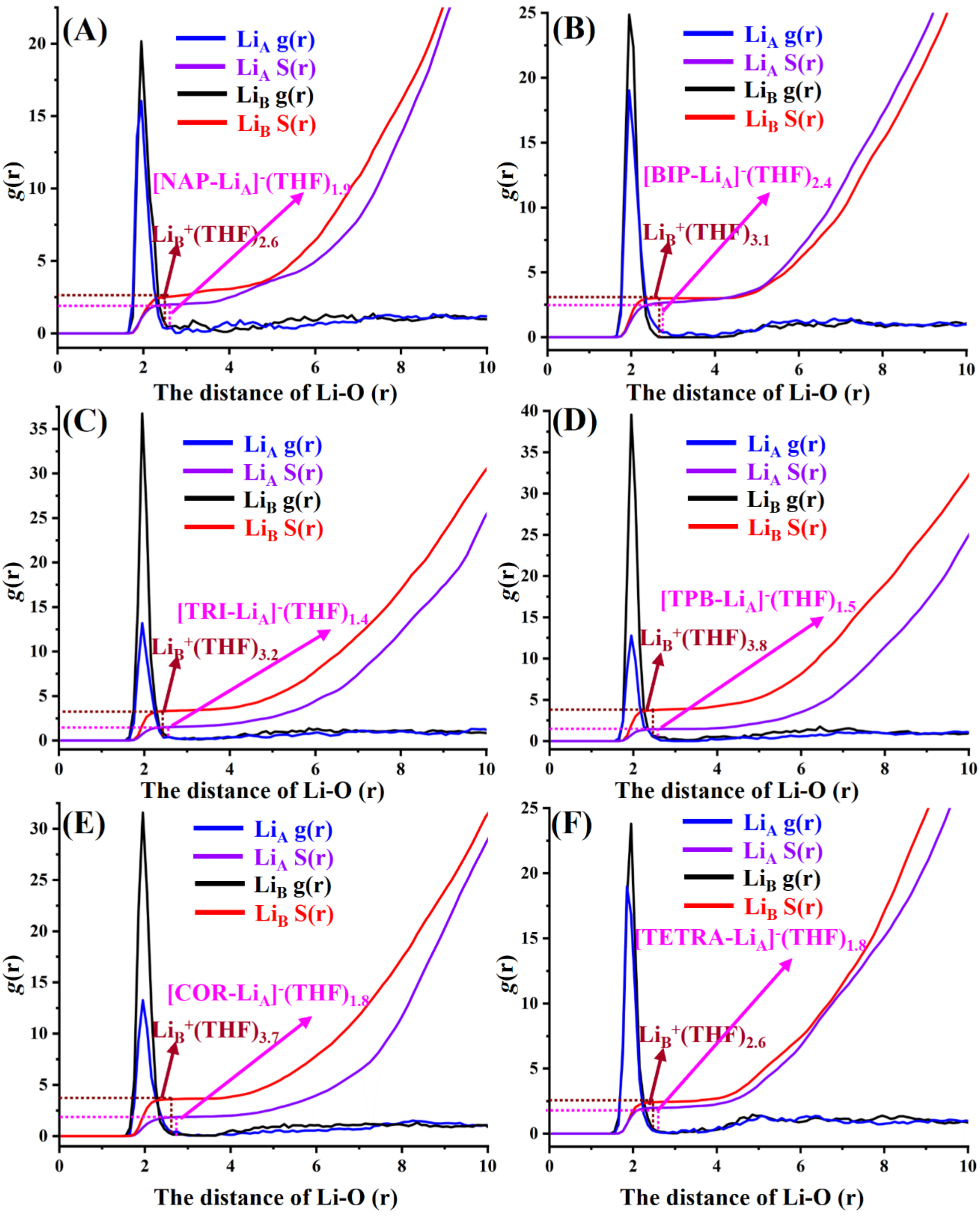

Figure S10. Radial distribution functions (g(r), black and blue curves) of Li-O_{THF} and their integrals for coordination numbers, showing the coordinating modes of Li^+ by O_{THF} for PAH-Li-SESs containing $[PAH\text{-}Li_A]^-(THF)_{1\text{-}2}$ and $Li_B^+(THF)_{2\text{-}4}$ with the $Li^+ \leftarrow O_{THF}$ dative bonds where PAH=NAP, BIP, TRI, TPB, COR, and TETRA, respectively.

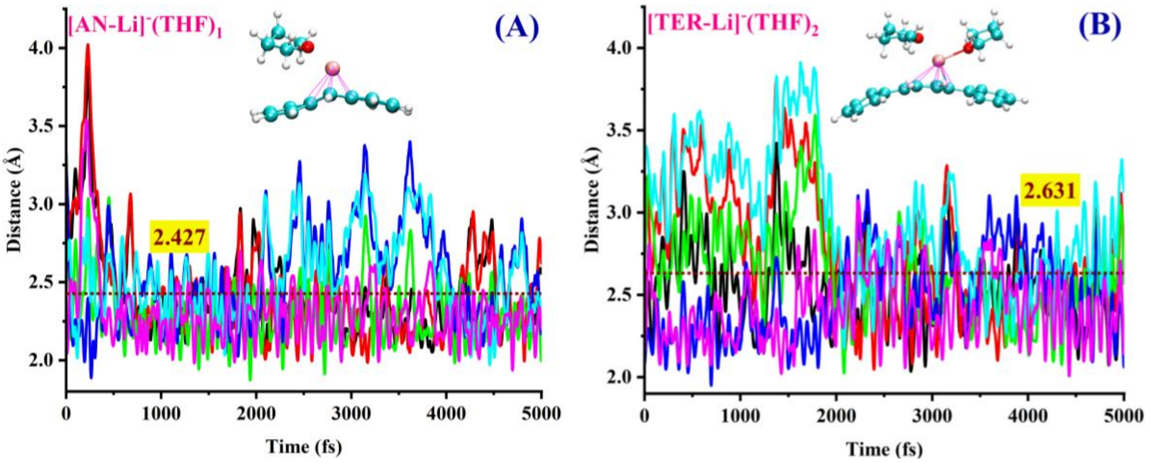

Figure S11. The distance evolutions of Li^+···C (in the Li^+ interacted benzene ring) in [AN-Li]$^-$(THF)$_1$ (**A**) and [TER-Li]$^-$(THF)$_2$ (**B**).

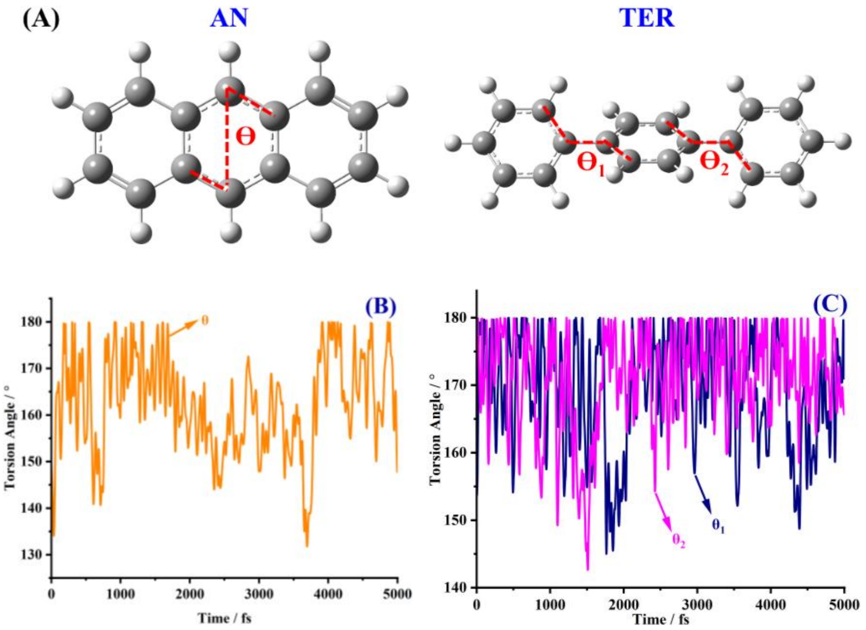

Figure S12. (A) Schematic diagram of twisting angles for PAHs in THF (AN/left: ϴ = 180°; TER/right: $ϴ_1 = ϴ_2$ = 143.1°). **(B)** Variation tendency of torsion angle for AN in AN-Li-SESs as the change of time (0 - 5 ps) extracted from the AIMD trajectory. The average value of twisting angle (ϴ) for AN-SME is 162.4°, displaying that distortion of AN is due to the binding of AN and Li^+/e^-_{sol}. **(C)** Variation tendencies of torsion angle for TER in TER-Li-SESs solution as the change of time (0 - 5 ps) extracted from the AIMD trajectory. The twisting angles ($ϴ_1$ and $ϴ_2$) for TER-SME are 168.9° and 170.6°, respectively, showing that distortion of TER is due to the binding of TER and Li^+/e^-_{sol}.

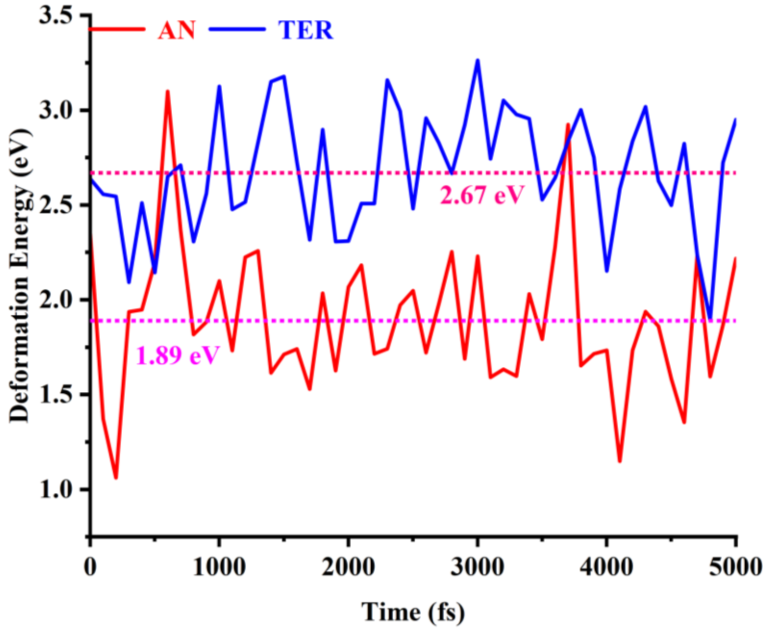

Figure S13. Time evolutions of the average deformation energies ($\Delta E_{\mathrm{def}} = E_{\mathrm{PAH(PAH\text{-}2Li@THF)}} - E_{\mathrm{PAH(THF)}}$) of PAHs in the AIMD trajectories of Li-SESs containing AN-SME and TER-SME, respectively.

Table S3. LUMO Energies of the Optimized PAH Molecules (the gaseous ones, E_{LUMO}(PAH-opt)) and the Deformed Structures of PAH Molecules in Their SMEs (E_{LUMO}(PAH-def)). $\Delta E_{\mathrm{LUMO}} = E_{\mathrm{LUMO}}$(PAH-def) – E_{LUMO}(PAH-opt), which reflect the deformation effect due to the formation of SMEs. All are in eV.

PAHs	E_{LUMO}(PAH-opt)	E_{LUMO}(PAH-def)	ΔE_{LUMO}
TETRA	-2.855	-3.125	-0.270
COR	-1.943	-2.836	-0.893
AN	-1.886	-2.643	-0.777
TER	-1.418	-2.250	-0.832
TRI	-1.223	-2.008	-0.785
NAP	-1.246	-2.047	-0.801
TPB	-1.178	-1.828	-0.650
BIP	-1.003	-1.726	-0.723

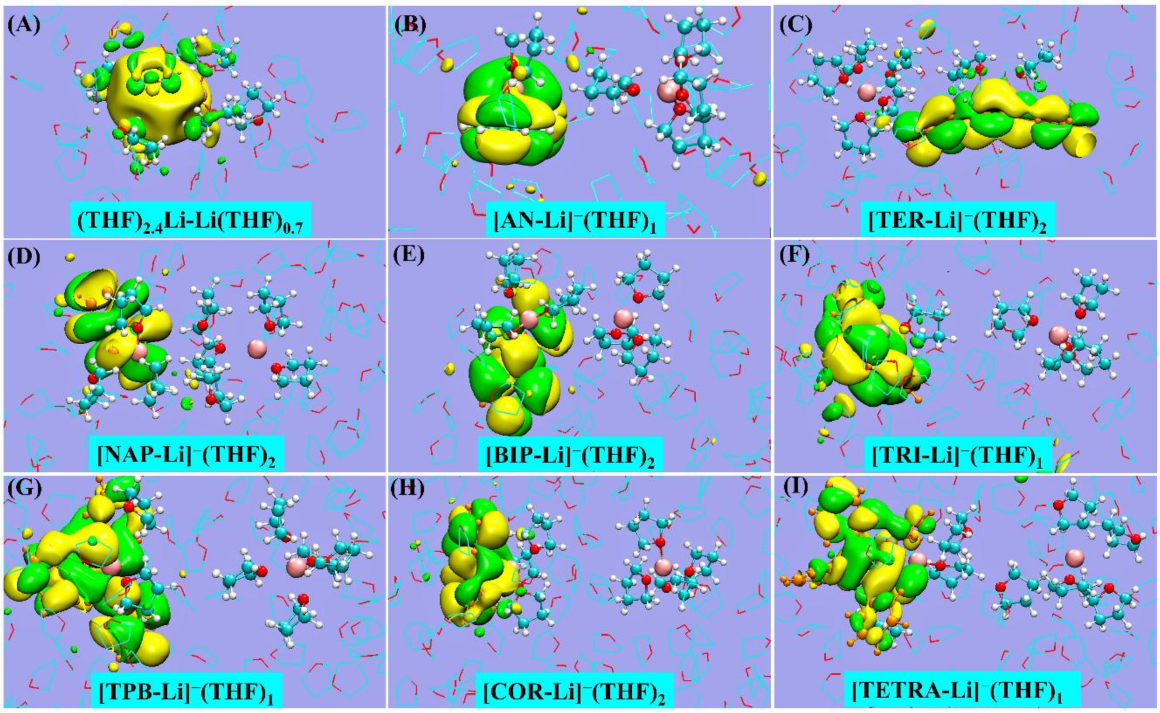

Figure S14. HOMO distributions (isovalue = 0.01) of some representative snapshots at arbitrary times extracted from the Li_2@THF **(A)** and PAH-Li-SESs (PAH=AN, TER, NAP, BIP, TRI, TPB, COR and TETRA) **(B-I)** trajectories, respectively. HOMO indicates that two electrons in Li_2@THF localize at Li_2 dimer, forming novel $(THF)_{2.4}$Li-Li$(THF)_{0.7}$ supermolecule entity. For PAH-Li-SESs, two electrons localize at PAH, forming the [PAH-Li]$^-$$(THF)_n$ (n=1-2) SMEs, e.g. [AN-Li]$^-$$(THF)_1$ or [TER-Li]$^-$$(THF)_2$.

Table S4. Dipole Moments (Debye) of the SMEs with and without THF Ligands.

SMEs	Dipole Moments	[PAH-Li]$^-$	Dipole Moments
[AN-Li]$^-$$(THF)_1$	55.33	[AN-Li]$^-$	52.09
[TER-Li]$^-$$(THF)_2$	89.92	[TER-Li]$^-$	89.73
[NAP-Li_A]$^-$$(THF)_2$	94.42	[NAP-Li_A]$^-$	86.88
[BIP-Li_A]$^-$$(THF)_2$	88.53	[BIP-Li_A]$^-$	86.94
[TRI-Li_A]$^-$$(THF)_1$	52.67	[TRI-Li_A]$^-$	50.52
[TPB-Li_A]$^-$$(THF)_1$	59.62	[TPB-Li_A]$^-$	56.48
[COR-Li_A]$^-$$(THF)_2$	62.29	[COR-Li_A]$^-$	61.27
[TETRA-Li_A]$^-$$(THF)_1$	49.54	[TETRA-Li_A]$^-$	45.65

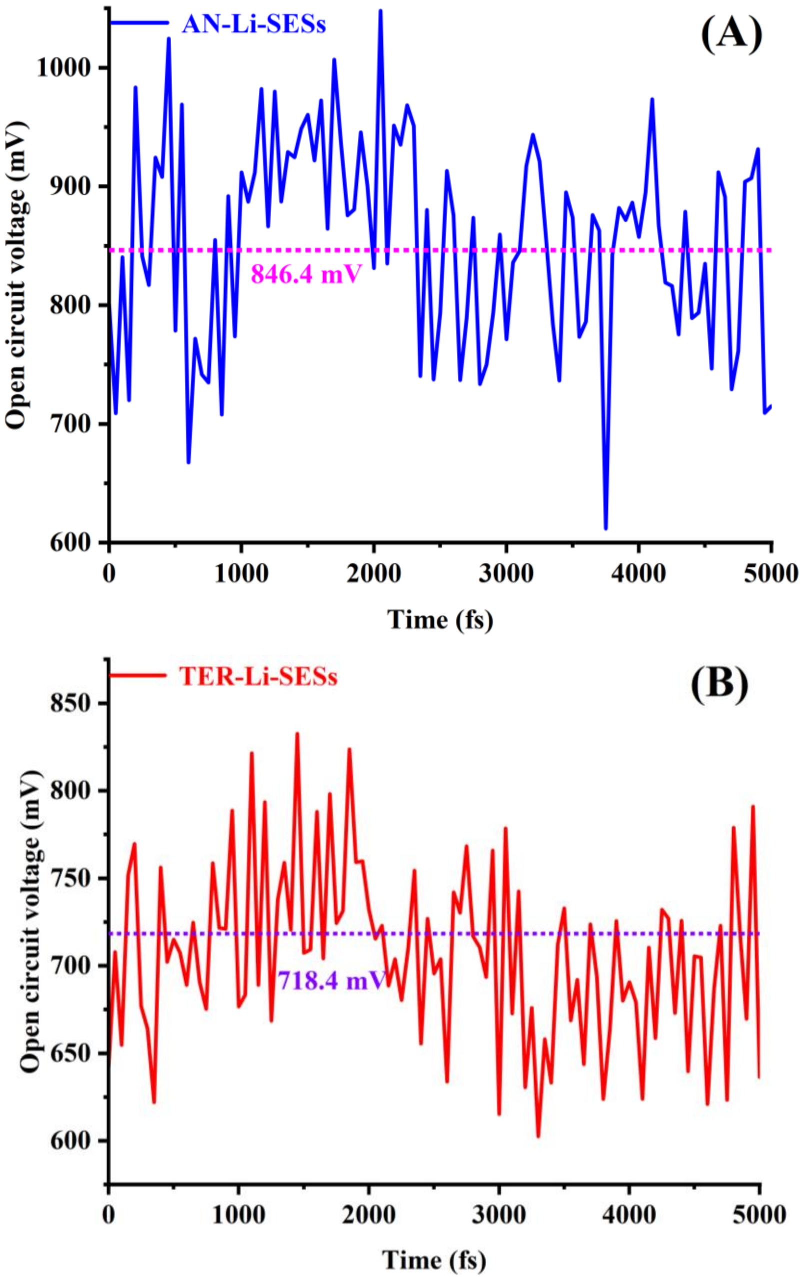

Figure S15. Time evolutions of predicted OCVs for AN-Li-SESs and TER-Li-SESs and the average values for them are 846.4 mV and 718.4 mV, respectively.

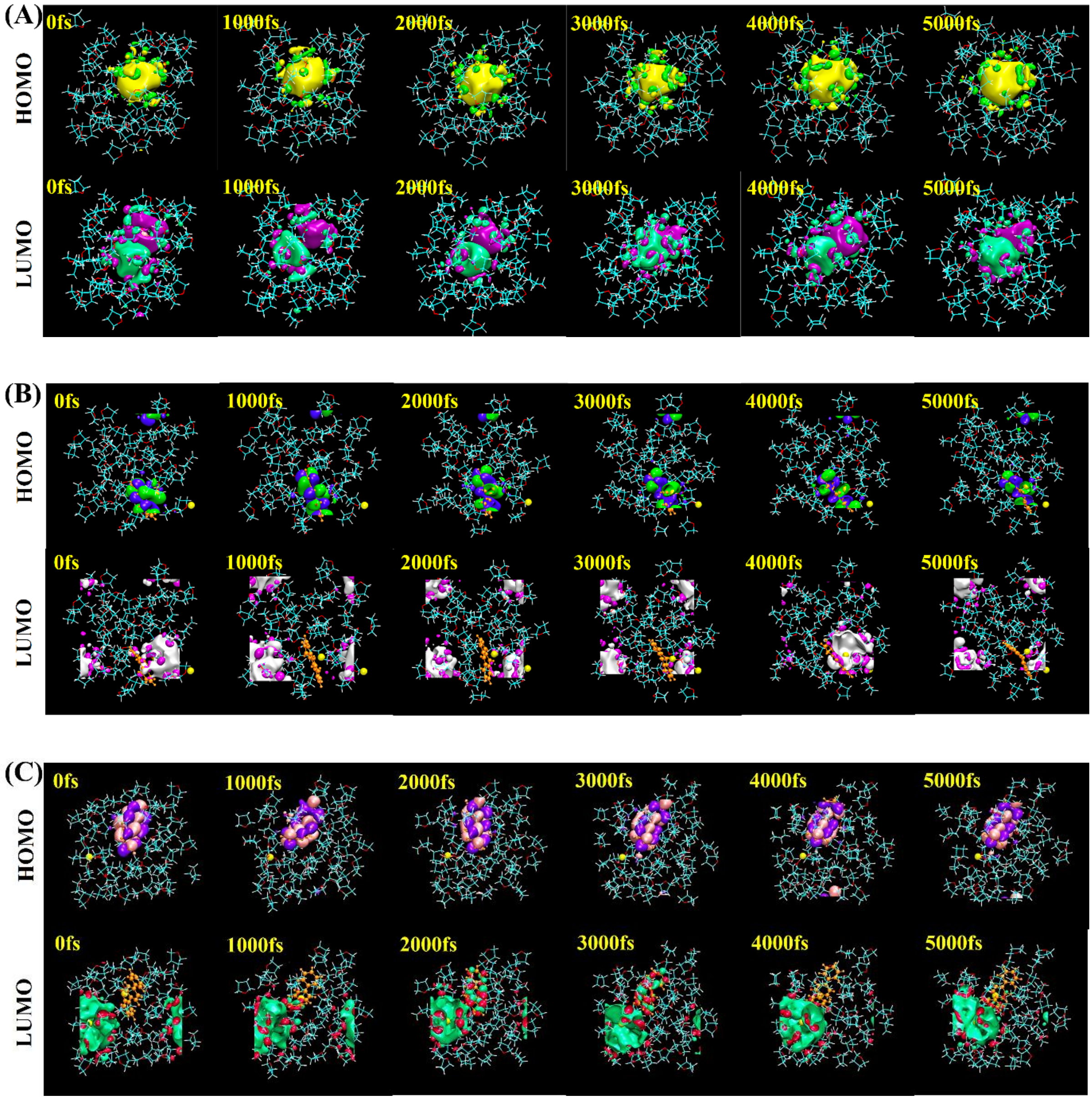

Figure S16. HOMO and LUMO distributions (isovalue = 0.01) of some representative snapshot at different times extracted from the Li_2@THF trajectory **(A)**, AN-Li-SESs trajectory **(B)** and TER-Li-SESs trajectory **(C)**, respectively. The two electrons in the Li_2@THF system localize in Li_2 dimer, forming novel $(THF)_{2.4}$Li-Li$(THF)_{0.7}$ supermolecule entity, and LUMO distributions corresponds to the Li-Li anti-bonding orbitals of $(THF)_{2.4}$Li-Li$(THF)_{0.7}$ entities. For the Li-SESs with a PAH (AN or TER) molecule, two electrons localize in PAH molecule, resulting in the formation of AN-SME or TER-SME supermolecule entities.

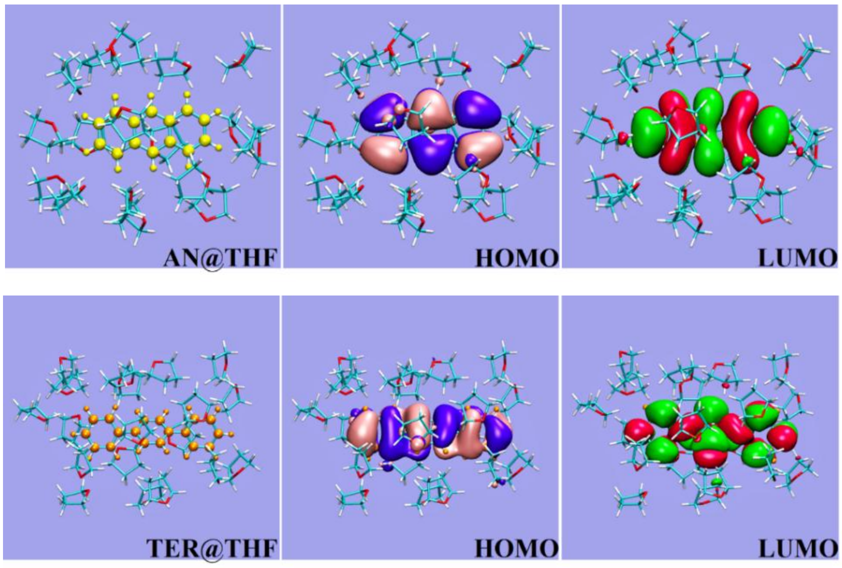

Figure S17. The structures and HOMO and LUMO distributions (isovalue = 0.01) of representative snapshot configurations extracted from AN@THF and TER@THF trajectories.

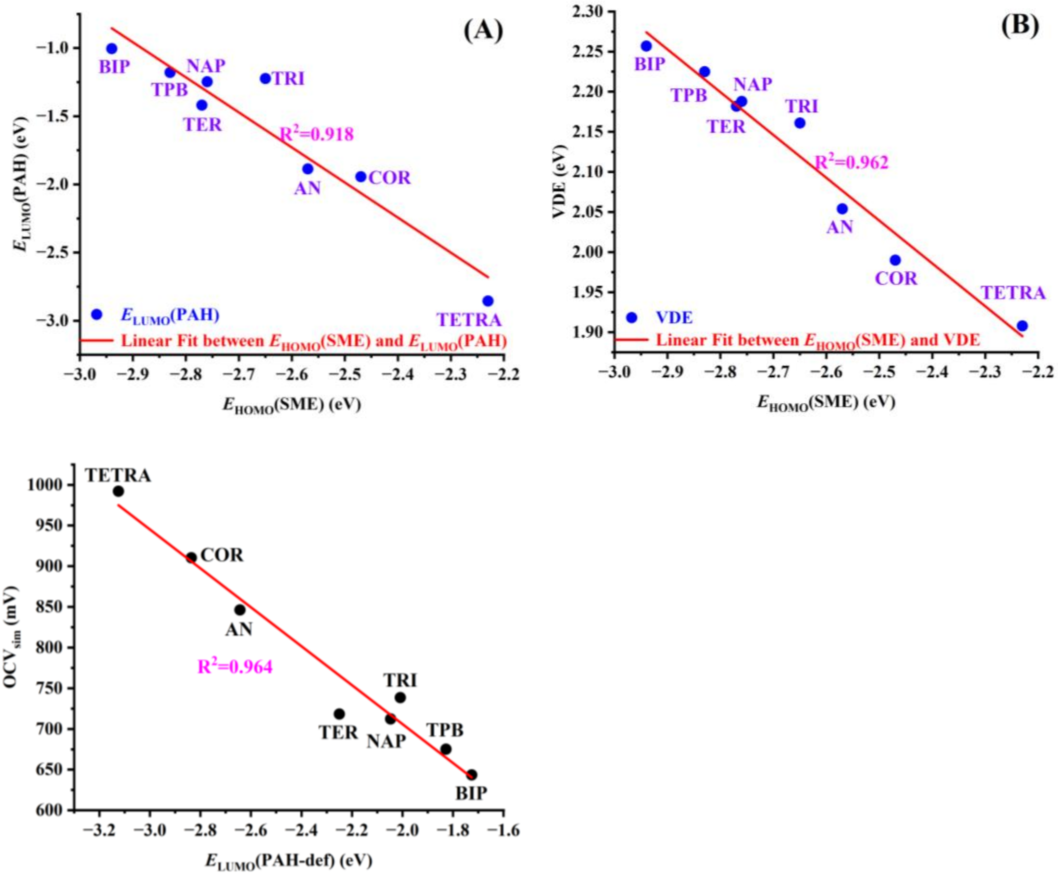

Figure S18. Correlations of E_{HOMO}(SME) with E_{LUMO}(PAH) and first VDE of SMEs and that between OCV_{siml} and E_{LUMO}(PAH-def) for eight PAH-Li-SESs where PAH-def denotes the PAH configuration in SME.

5. Direct Predictions of OCVs for Potential PAH-Li-SESs with Unexplored PAH Additive Molecules and Reliability Analyses

Utilizing this correlation (the fitting equation: OCV_{pred} = -194.17 E_{LUMO}(PAH-opt) + 470.15 fitted over the AIMD-simulated OCV_{siml} of 8 PAH-Li-SESs), we further predict the OCVs of the corresponding PAH-Li-SESs for some additional **15** PAH additive molecules (unexplored), and all the results are given in Table S5, together with the corresponding AIMD-simulated OCV_{siml} (green-highlighted) and experimental values (blue-highlighted) if available. The comparison among them is displayed in **Figure S19** which shows the high agreement.

Table S5. Structures, HOMO/LUMO and LUMO Energies of the Optimized Additive Molecules (PAHs) and Their Corresponding OCVs Predicted Using E_{LUMO}(PAH-opt) via the Fitting Equation (**pred,** OCV = -194.17 E_{LUMO}(PAH-opt) + 470.15 fitted over the AIMD-simulated OCVs of 8 molecules marked by ※). The OCV Values in Round Brackets Denote the Direct AIMD-Simulated Results Using VDEs of SMEs (**siml**)/(**siml**) in which the green ones participate in the fitting equation and the blue ones don't and Those in Square Brackets Are the Experiment Ones Available (**expt**).

**PAHs**	**Structures**	**HOMOs**	**LUMOs**	**E_{LUMO} (PAH-opt) eV**	**OCV/mV Pred (siml)/(siml) [expt]**
2,3,5,6-tetraphenyl-*p*-benzoquinone (**TPBQ**)	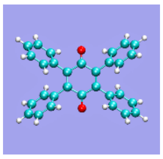	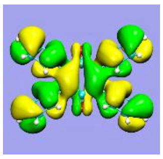	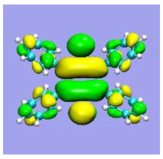	-3.497	1149.2 (1153.4)
2,3,5,6-tetraethyl-*p*-benzoquinone (**TEBQ**)	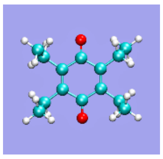	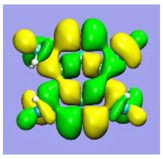	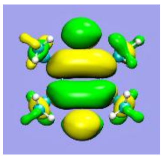	-3.322	1115.2 (1049.8)
2,3,5,6-tetraphenyl-*p*-benzoquinone (Duroquinone) (**TMBQ**)	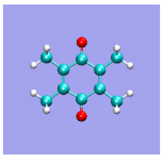	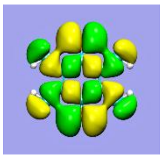	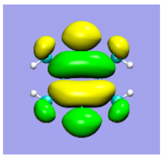	-3.315	1113.8

Diazapentacene (**DAPc**)	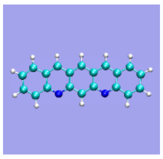	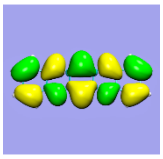	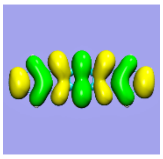	-3.174	1086.5
※ Tetraphenyl-cyclopentadienone (**TETRA-Ph-O**)	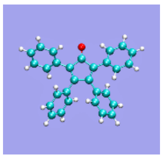	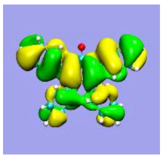	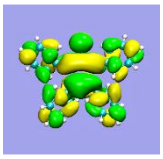	-2.855	1024.5 (992.3)
Pentacene (**Pc**)	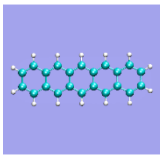	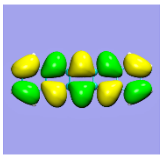	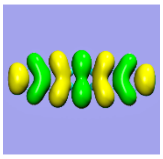	-2.747	1003.5 (985.4)
2,3,4,5-tetraphenyl-cyclopentadienimine (**TETRA-Ph-N**)	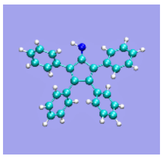	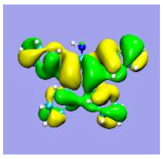	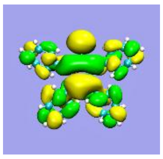	-2.639	982.6
2,3,4,5-tetraethyl-cyclopentadienone (**TETRA-Et-O**)	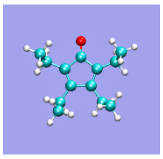	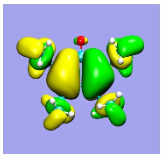	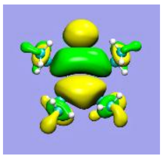	-2.488	953.3 (944.2)
Tetracene (**Tc**)	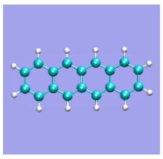	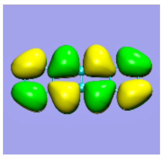	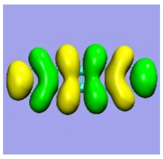	-2.448	945.5
2,3,4,5-tetramethyl-cyclopentadienone (**TETRA-Me-O**)	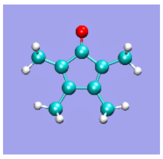	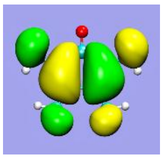	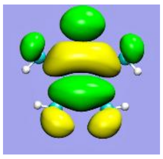	-2.406	937.3
Hexa-*peri*-hexabenzocoronene (**HBC**)	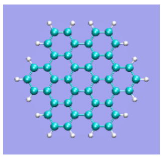	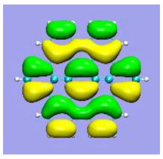	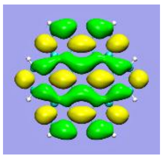	-2.021	862.6
※ Corannulene (**COR**)	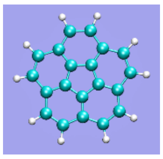	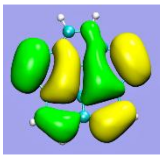	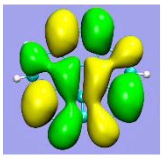	-1.944	847.6 (910.5)
※ Anthracene (**AN**)	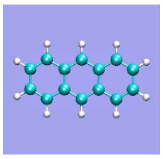	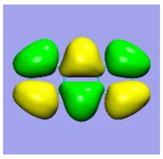	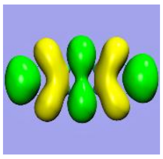	-1.887	836.6 (846.4) [900.0]
Coronene (**CORo**)	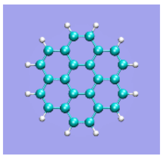	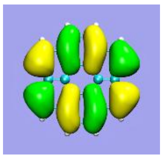	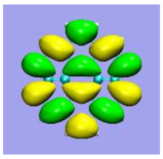	-1.777	815.2

3-phenylpyridine<br>(**3-PPy**)	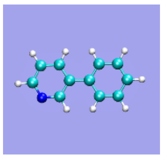	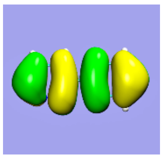	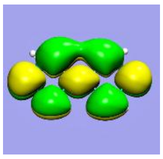	-1.652	790.9<br>(749.4)
4-phenylpyridine<br>(**4-PPy**)	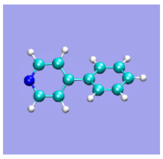	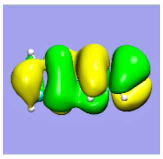	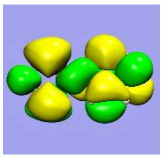	-1.613	783.4
2-phenylpyridine<br>(**2-PPy**)	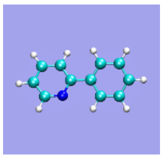	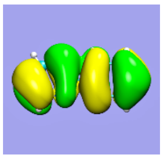	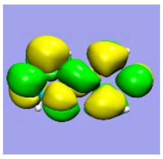	-1.506	762.6
※ *p*-terphenyl<br>(**TER**)	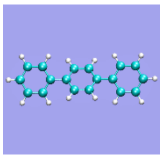	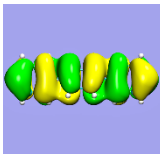	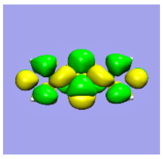	-1.418	745.5<br>(718.4)<br>[725.0]
※ Naphthalene<br>(**NAP**)	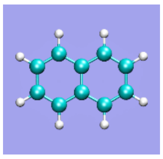	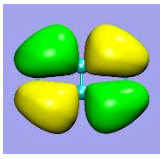	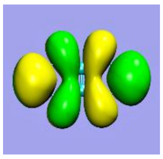	-1.246	712.1<br>(712.4)<br>[720.0]
※ Triphenylene<br>(**TRI**)	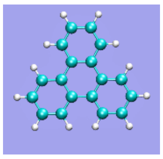	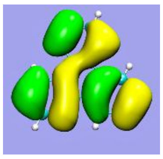	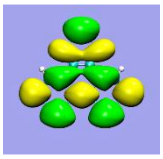	-1.223	707.6<br>(738.6)<br>[747.2]
※ 1,3,5-triphenyl-<br>benzene<br>(**TPB**)	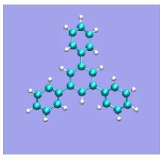	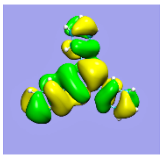	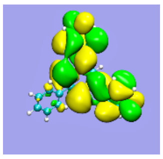	-1.179	699.1<br>(675.2)<br>[700.0]
Hexaphenylbenzene<br>(**HPB**)	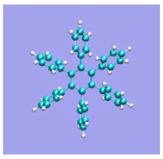	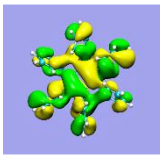	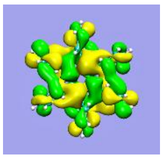	-1.138	691.12
※ Biphenyl (**BIP**)	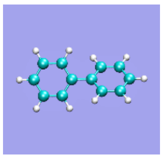	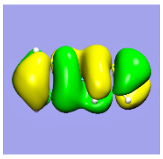	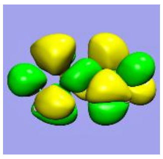	-1.073	678.5<br>(643.5)<br>[680.0]

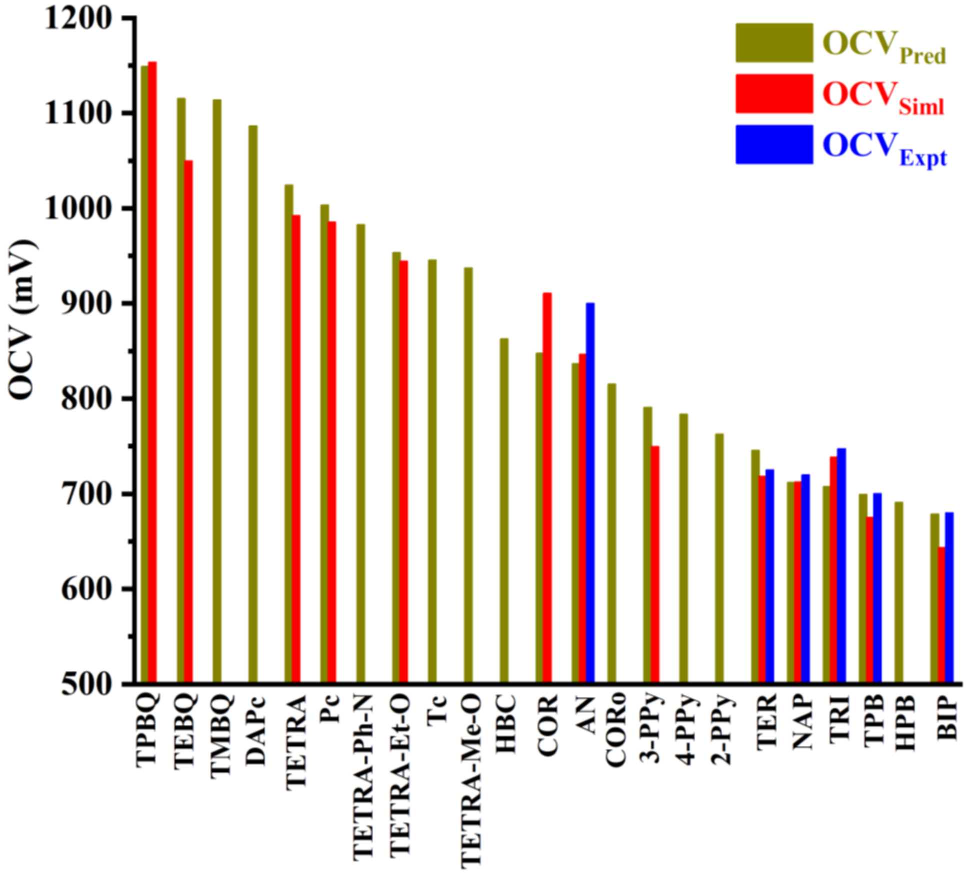

Figure S19. Comparison of OCV results (**Table S5**) among the AIMD-simulated OCV_{siml}, OCV_{pred} predicted from the fitting equation of OCV_{siml}~E_{LUMO}(PAH) and experimental OCV (OCV_{expt}) for all 23 considered PAH additives.

The Prediction Accuracy Analyses

To further confirm the reliability of our predicted OCVs, we also predict the OCVs using the correlation of OCV_{expt}~E_{LUMO}(PAH): OCV_{pred}(A) = -245.836 E_{LUMO}(PAH) + 419.469, i.e. the fitting equation from the experimental OCV values of PAH-Li-SESs with 6 PAH molecules for which the experimental OCVs are available (**Table S6**, the top table). Table S6 indicates that the predicted OCVs (OCVpred(A)) are very close to the original experimental ones with minor errors (Δ_1), and the deviation percentages ($|P_1|$) are smaller than 6%.

We also make a similar evaluation on the predictions using the fitting equation from the AIMD-simulated OCV_{siml}: OCV_{pred}(B) = -194.17 E_{LUMO}(PAH) + 470.15, i.e. the fitting equation from the AIMD-simulated OCV values of PAH-Li-SESs with 8 PAH molecules (**Table**

S6, the bottom table). The deviations between the AIMD-simulated OCV_{siml} and $OCV_{pred}(B)$ predicted by the fitting equation are also extremely small with the relative percentage $|P_2| < 5.4\%$. In particular, compared with the experimental OCV_{expt}, the deviation percentage $|P_3| < 7\%$.

In short, all these analyses indicate that this linear correlation (OCV_{siml}~E_{LUMO}(PAH)) has quite high predicting ability.

To further confirm the predicting ability, we give all results predicted from two fitting equations obtained from the AIMD-simulated OCV_{siml} and experimental OCV_{expt} in **Table S7**. **Figure S20** indicates high correlation between them.

Table S6. The experimental, simulated and predicted OCVs using different fitting equations and their variation percentages. All OCVs are in mV.

**PAH**	OCV_{expt}	$OCV_{pred}(A)$	Δ_1 ($\Delta_1 = OCV_{pred}(A) - OCV_{expt}$)		P_1 ($P_1 = \Delta_1/OCV_{expt}$)	
	The fitted equation from OCV_{expt}: $OCV_{pred}(A) = -245.836\ E_{LUMO}(PAH) + 419.469$ (the expt-fitting equation **A**) where PAH is a gaseous molecule.					
**AN**	900	883.4	-16.6		-1.84%	
**TER**	725	768.1	43.1		5.94%	
**NAP**	720	725.8	5.8		0.81%	
**BIP**	680	683.3	3.3		0.49%	
**TRI**	747	720.1	-26.9		-3.60%	
**TPB**	700	709.3	9.3		1.33%	
**PAH**	OCV_{siml}	$OCV_{pred}(B)$	Δ_2	P_2	Δ_3	P_3
	The fitted equation from OCV_{siml}: $OCV_{pred}(B) = -194.17\ E_{LUMO}(PAH) + 470.15$ (the siml-fitting equation **B**) where PAH is a gaseous molecule.					
**AN**	846.4	836.6	-9.8	1.2%	-63.4	-7.0%
**TER**	718.4	745.5	27.1	3.8%	20.5	2.8%
**NAP**	712.4	712.1	-0.3	0.04%	-7.9	-1.1%
**BIP**	643.5	678.5	35.0	5.4%	-1.5	-0.2%
**TRI**	738.6	707.6	-31.0	4.2%	-39.4	-5.3%
**TPB**	675.2	699.1	23.9	3.5%	-0.9	-0.1%

Notes: The deviations $\Delta_2 = OCV_{pred}(B) - OCV_{siml}$ and $\Delta_3 = OCV_{pred}(B) - OCV_{expt}$ and the variation percentages $P_2 = \Delta_2/OCV_{siml}$ and $P_3 = \Delta_3/OCV_{expt}$.

Table S7. Prediction of OCVs on 23 Systems without OCV_{expt} and Comparison of OCV_{pred} results between two methods. The blue-highlighted values in the last column are the AIMD-simulated results for the verification of OCV_{pred} and the other values (green-highlighted) are used for fitting equation (eq.4). OCV_{pred}(A) are obtained from the fitting equation of OCV_{expt}~E_{LUMO}(PAH) over 6 PAH-Li-SES, while OCV_{pred}(B) are obtained from the fitting equation of OCV_{siml}~E_{LUMO}(PAH) over 8 PAH-Li-SESs.

**PAHs**	**OCV_{pred}(A)** (the expt-fitting equation)	**OCV_{pred}(B)** (the AIMD-simulated fitting equation)	**OCV_{siml}** (AIMD-simulated)
**TPBQ**	1279.16	1149.2	1153.4
**TEBQ**	1236.14	1115.2	1049.8
**TMBQ**	1234.42	1113.8	
**DAPc**	1199.75	1086.5	
**TETRA**	1121.33	1024.5	992.3
**Pc**	1094.78	1003.5	985.4
**TETRA-Ph-N**	1068.23	982.6	
**TETRA-Et-O**	1031.11	953.3	944.2
**Tc**	1021.28	945.5	
**TETRA-Me-O**	1010.95	937.3	
**HBC**	916.30	862.6	
**COR**	897.37	847.6	910.5
**AN**	883.36	836.6	846.4
**CORo**	856.32	815.2	
**3-PPy**	825.59	790.9	749.4
**4-PPy**	816.01	783.4	
**2-PPy**	789.69	762.6	
**TER**	768.06	745.5	718.4
**NAP**	725.78	712.1	712.4
**TRI**	720.13	707.6	738.6
**TPB**	709.31	699.1	675.2
**HPB**	699.23	691.1	
**BIP**	683.25	678.5	643.5

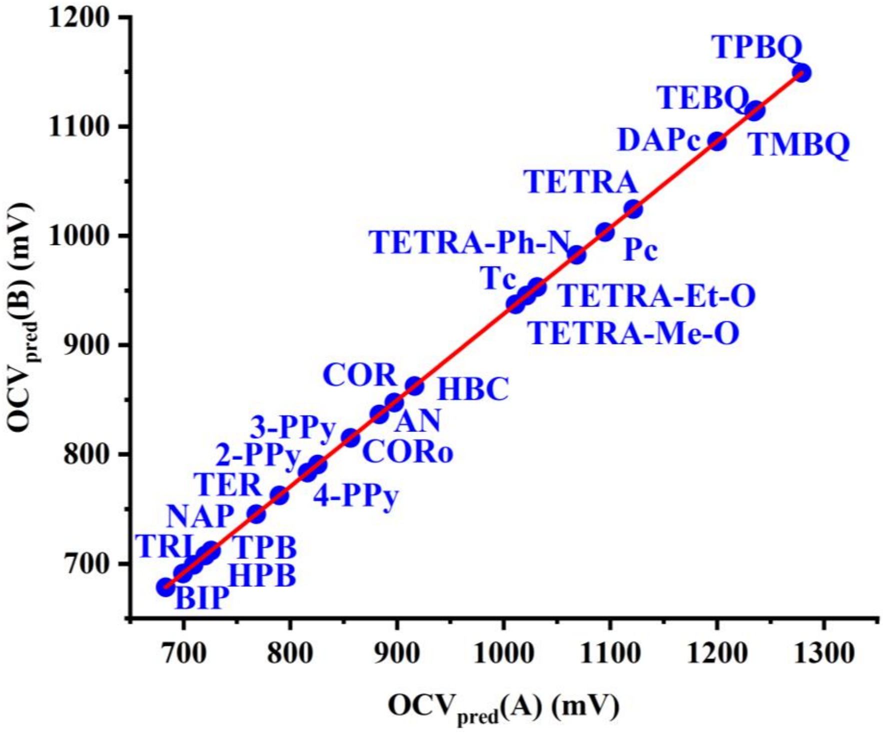

Figure S20. High linear correlation between the OCV$_{pred}$(A) predicted by the expt-fitting equation and OCV$_{pred}$(B) predicted by the AIMD-simulated fitting equation for 17 systems without OCV$_{expt}$ over all 23 systems.

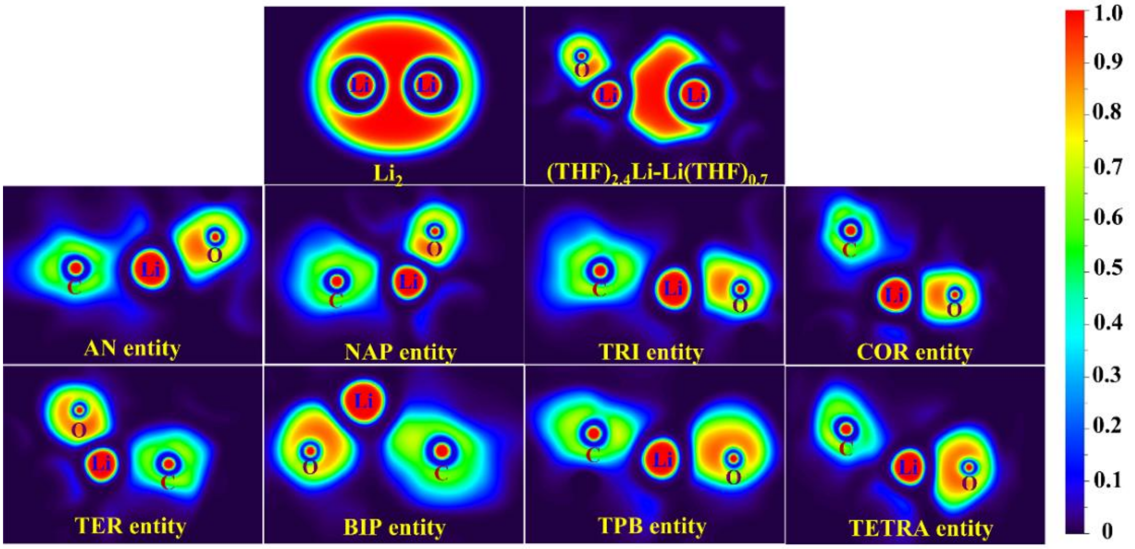

Figure S21. ELF of the representative entities for Li_2@THF system and PAH-Li-SESs systems in liquid THF, calculated at the B3LYP/6-311++G(d,p) level of theory.

6. Additional Trajectories Support the Conclusion

It is noteworthy that in AIMD simulation studies, appropriately increasing the number of simulation trajectories generally helps mitigate the influence of random fluctuations on the results, thereby improving the reliability and representativeness of the simulated data. However, in practice, the choice of trajectory count must strike a balance between computational resource availability and time constraints. To further validate the findings of this study, additional independent AIMD simulations were performed for both the Li_2@THF and TER-Li-SESs systems.

*g(*r) derived from the supplementary trajectory of the Li_2@THF system is presented in **Figure S22**, confirming the reproducibility of the observed asymmetric clusters and supporting their structural plausibility. For TER-Li-SESs system, the results from the additional trajectory, including *g*(r), variations in Li-Li and Li-O_{THF} distances, Mulliken charge distribution, and SOMO distributions (isovalue = 0.01), are shown in **Figure S23**, consistently reinforcing the analyses and discussions provided in the main text.

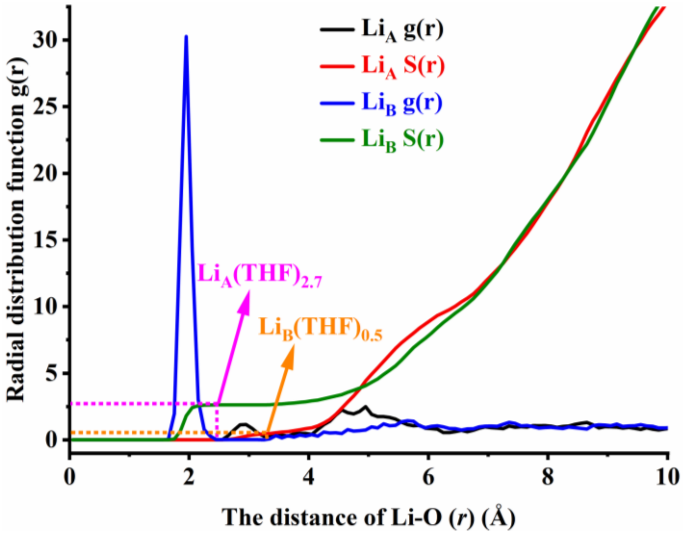

Figure S22. Radial distribution functions (*g*(r)) of Li-O_{THF} and their integrals for coordination numbers ($Li_A(THF)_{2.7}$, $Li_B(THF)_{0.5}$).

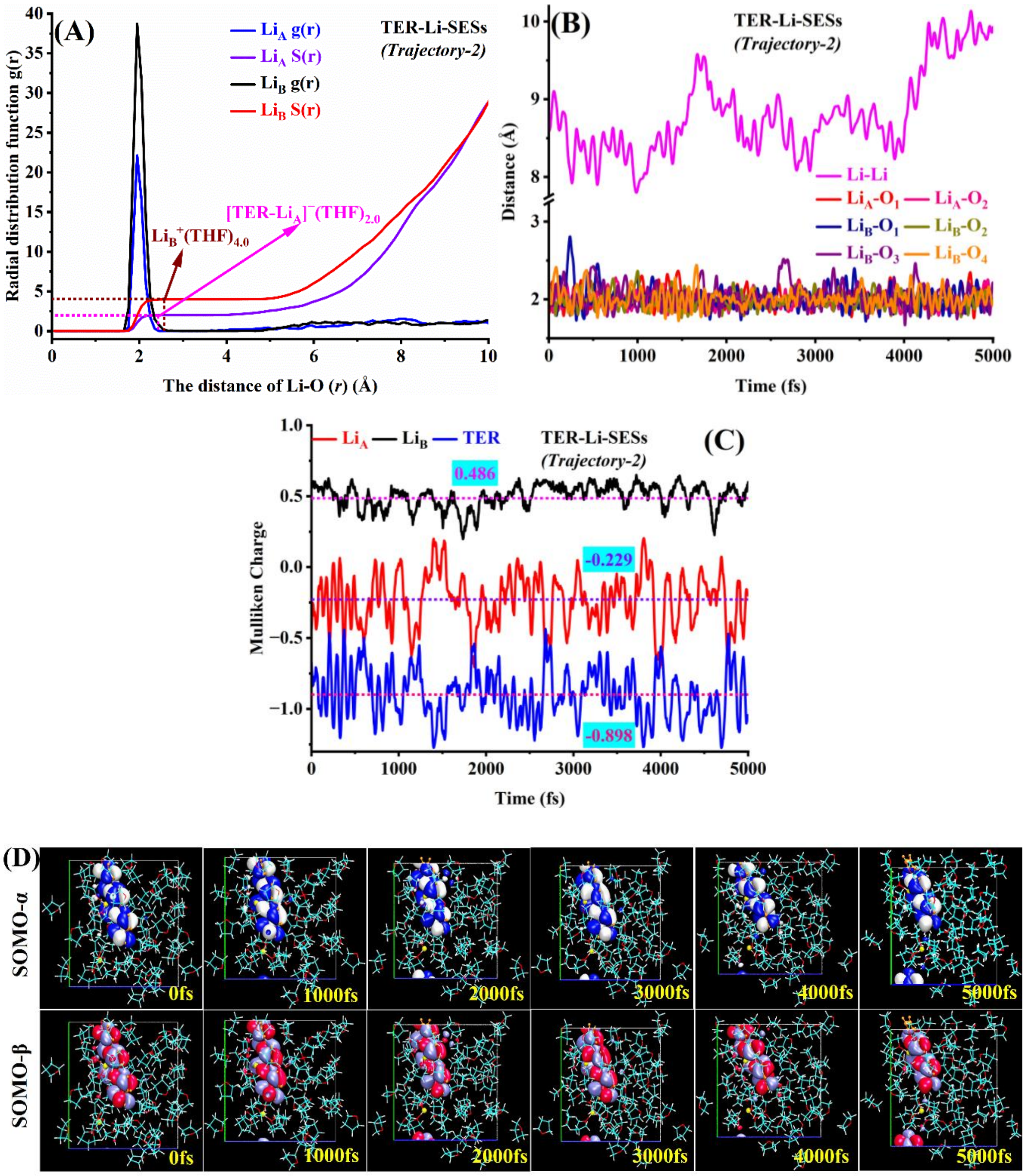

Figure S23. (A) Radial distribution functions, g(r) (black and blue lines), showing the coordinating modes of Li^+ by O_{THF} for TER-Li-SESs containing $[TER\text{-}Li]^-(THF)_2$ with the Li^+-O_{THF} dative bonds. (B) Time evolutions of $Li^+\cdots O$ distance between Li^+ and its near THF and Li···Li distance for TER-Li-SES. (C) Mulliken charge fluctuations of Li_A, Li_B and TER in TER-Li-SESs during 5 ps AIMD trajectories. (D) SOMO-α and SOMO-β distributions (isovalue = 0.01) of some representative snapshot at different times extracted from the dynamic trajectories of TER-Li-SESs.

7. References

(1) ***a***) Tan, K. S.; Grimsdale, A. C.; Yazami, R. *J. Phys. Chem. B.* **2012**, *116*, 9056-9060;

b) Tan, K. S.; Yazami, R. *Electrochim. Acta.* **2015**, *180*, 629-635;

c) Lunchev, A. V.; Liu, Z. H.; Su, H. B.; Yazami, R.; Grimsdale, A. C. *Electrochim. Acta.* **2018**, *292*, 142-146;

d) Tan, K. S.; Lunchev, A. V.; Stuparu, M. C.; Grimsdale, A. C.; Yazami, R. *J. Vis. Exp.* **2016**, *116*, e54366;

e) Lunchev, A. V.; Tan, K. S.; Grimsdale, A. C.; Yazami, R. *New J. Chem.* **2018**, *42*, 15678-15683;

f) Liu, Z. H.; Lunchev, A. V.; Li, W.; Ruan, S. C.; Yazami, R.; Grimsdale, A. C.; Su, H. B. *ACS. Omega.* **2019**, *4*, 4707-4711;

g) Lunchev, A. V.; Liu, Z. H.; Li, W.; Ruan, S. C.; Su, H. B.; Yazami, R.; Grimsdale, A. C. *J. Electroanal. Chem.* **2020**, *875*, 114148;

h) Lim, Z. B.; Tan, K. S.; Lunchev, A. V.; Li, H. R.; Cho, S. J.; Grimsdale, A. C.; Yazami, R. *Synthetic Metals* **2015**, *200*, 85-90;

i) Tan, K. S.; Grimsdale, A. C.; Yazami, R. *Sci. Rep.* **2017**, *7,* 6502.

(2) Rinaldi, A.; Tan, K. S.; Wijaya, O.; Wang, Y.; Yazami, R. *Advances in batteries for large- and medium-scale energy storage applications in power systems and electric vehicles.* Woodhead Publishing Ltd. **2014**, pp. 387-440.

(3) Rinaldi, A.; Y. Wang, Y.; Tan, K. S.; Wijaya, O.; Yazami, R. *Advances in batteries for medium and large-scale energy storage.* Woodhead Publishing. **2015**, pp. 387-440.

(4) ***a***) Lee, J. H.; Lee, H.; Lee, J.; Kang, T. W.; Park, J. H.; Shin, J. H.; Lee, H.; Majhi, D.; Lee, S. U.; Kim, J-H. *ACS Nano* **2023**, 17, 17372-17382;

b) Shankar, U.; Gogoi, R.; Sethi, S. K.; Verma, A. *In Forcefields for Atomistic-Scale Simulations: Materials and Applications,* Springer, **2022**; pp 299-313.

(5) Kühne, T. D.; Iannuzzi, M.; Ben, M. D.: Rybkin, V. V.; Seewald, P.; Stein, F.; Laino, T.; Khaliullin, R. Z.; Schütt, O.; Schiffmann, F.; Golze, D.; Wilhelm, J.; Chulkov, S.; Bani-Hashemian, M. H.; Weber, V.; Borštnik, U.; Taillefumier, M.; Jakobovits, A. S.; Lazzaro, A.; Pabst, H.; Müller, T.; Schade, R.; Guidon, M.; Andermatt, S.; Holmberg, N.; Schenter,

G. K.; Hehn, A.; Bussy, A.; Belleflamme, F.; Tabacchi, G.; Glöß, A.; Lass, M.; Bethune, I.; Mundy, C. J.; Plessl, C.; Watkins, M.; VandeVondele, J.; Krack, M.; Hutteret, J. *J. Chem. Phys.* **2020**, 19, 194103.

(6) Frisch, M. J.; Trucks, G. W.; Schlegel, H. B.; Scuseria, G. E.; Robb, M. A.; Cheeseman, J. R.; Scalmani, G.; Barone, V.; Petersson, G. A.; Nakatsuji, H.; Li, X.; Caricato, M.; Marenich, A. V.; Bloino, J.; Janesko, B. G.; Gomperts, R.; Mennucci, B.; Hratchian, H. P.; Ortiz, J. V.; Izmaylov, A. F.; Sonnenberg, J. L.; Williams; Ding, F.; Lipparini, F.; Egidi, F.; Goings, J.; Peng, B.; Petrone, A.; Henderson, T.; Ranasinghe, D.; Zakrzewski, V. G.; Gao, J.; Rega, N.; Zheng, G.; Liang, W.; Hada, M.; Ehara, M.; Toyota, K.; Fukuda, R.; Hasegawa, J.; Ishida, M.; Nakajima, T.; Honda, Y.; Kitao, O.; Nakai, H.; Vreven, T.; Throssell, K.; Montgomery Jr., J. A.; Peralta, J. E.; Ogliaro, F.; Bearpark, M. J.; Heyd, J. J.; Brothers, E. N.; Kudin, K. N.; Staroverov, V. N.; Keith, T. A.; Kobayashi, R.; Normand, J.; Raghavachari, K.; Rendell, A. P.; Burant, J. C.; Iyengar, S. S.; Tomasi, J.; Cossi, M.; Millam, J. M.; Klene, M.; Adamo, C.; Cammi, R.; Ochterski, J. W.; Martin, R. L.; Morokuma, K.; Farkas, O.; Foresman, J. B.; Fox, D. J. *Gaussian 16 Rev. C.01*, Wallingford, CT, **2016**.

(7) del Olmo, D.; Pavelka, M.; Kosek, J. *J. Non-Equil. Thermody.* **2021**, *46*, 91-108

(8) Goodenough, J. B.; Kim, Y. *Chem. Mater.* **2010**, *22*, 587-603.

(9) ***a***) Urban, A.; Seo, D-H.; Ceder, G. *npj Comput. Mater.* **2016**, *2*, 16002;

b) Aydinol, M. K.; Kohan, A. F.; Ceder, G.; Cho, K.; Joannopoulos, J. *Phys. Rev. B: Condens. Matter Mater. Phys.* **1997**, *56*, 1354-1365.

(10) Michaelson, H. B. *J. Appl. Phys.* **1977**, *48*, 4729-4733.

(11) Huang, S.; Gao, L.; Fu, Q.; Bu, Y. X. *J. Phys. Chem. Lett.* **2021**, *12*, 3274-3280.

(12) Methfessel, M.; Hennig, D.; Scheffler, M. *Phys. Rev. B: Condens. Matter Mater. Phys.* **1992**, *46*, 4816-4829.

(13) Skriver, H. L.; Rosengaard, N. M. *Phys. Rev. B: Condens. Matter Mater. Phys.* **1992**, *46*, 7157-7168.

(14) Stevanović, V.; Lany, S.; Ginley, D. S.; Tumas, W.; Zunger, A. *Phys. Chem. Chem. Phys.* **2014**, *16*, 3706-3714.

(15) Martyna, G. J.; Klein, M. L.; Tuckerman, M. *J. Chem. Phys.* **1992**, *97*, 2635-2643.

(16) ***a***) Perdew, J.; Burke, K.; Ernzerhof, M. *Phys. Rev. Lett.* **1996**, *77*, 3865-3868;

b) Grimme, S. *J. Comput. Chem.* **2006**, 15, 1787-1799.

(17) ***a***) Goedecker, S.; Teter, M.; Hutter, J. *Phys. Rev. B.* **1996**, *3*, 1703-1710;

b) Hartwigsen, C.; Goedecker, S.; Hutter, J. *Phys. Rev. B.* **1998**, *7*, 3641-3662.

(18) Lippert, G.; Hutter, J.; Parrinello, M. *Mol. Phys.***1997**, *92*, 477-488.

(19) Huang, H. B.; Xue, L. J.; Bu, Y. X. *Chem-Eur. J.* **2023**, *29*, e202302253.

(20) Becke, A. D. *J. Chem. Phys.* **1993**, *98*, 5648-5652.
